# Dentate Gyrus Granule Cells Show Stability of BDNF Protein Expression in Mossy Fiber Axons with Age, and Resistance to Alzheimer’s Disease Neuropathology in a Mouse Model

**DOI:** 10.1523/ENEURO.0192-23.2023

**Published:** 2024-03-01

**Authors:** Chiara Criscuolo, Elissavet Chartampila, Stephen D. Ginsberg, Helen E. Scharfman

**Affiliations:** ^1^Center for Dementia Research, The Nathan Kline Institute for Psychiatric Research, Orangeburg, NY 10962; ^2^Department of Child & Adolescent Psychiatry, NewYork University Grossman School of Medicine, New York, NY 10016; ^3^Department of Cell Biology and Physiology, University of North Carolina at Chapel Hill, Chapel Hill, NC 27599; ^4^Department of Neuroscience & Physiology, NewYork University Grossman School of Medicine, New York, NY 10016; ^5^Psychiatry, NewYork University Grossman School of Medicine, New York, NY 10016; ^6^NYU Neuroscience Institute, NewYork University Grossman School of Medicine, New York, NY 10016

**Keywords:** ΔFosB, amyloid-β, APPSwe, hippocampus, plaque, Tg2576

## Abstract

Brain-derived neurotrophic factor (BDNF) is important in the development and maintenance of neurons and their plasticity. Hippocampal BDNF has been implicated in Alzheimer’s disease (AD) because hippocampal levels in AD patients and AD animal models are often downregulated, suggesting that reduced BDNF contributes to AD. However, the location where hippocampal BDNF protein is most highly expressed, the mossy fiber (MF) axons of dentate gyrus granule cells (GCs), has been understudied, and not in controlled conditions. Therefore, we evaluated MF BDNF protein in the Tg2576 mouse model of AD. Tg2576 and wild-type (WT) mice of both sexes were examined at 2–3 months of age, when amyloid-β (Aβ) is present in neurons but plaques are absent, and 11–20 months of age, after plaque accumulation. As shown previously, WT mice exhibited high levels of MF BDNF protein. Interestingly, there was no significant decline with age in either the genotype or sex. Notably, MF BDNF protein was correlated with GC ΔFosB, a transcription factor that increases after 1–2 weeks of elevated neuronal activity. We also report the novel finding that Aβ in GCs or the GC layer was minimal even at old ages. The results indicate that MF BDNF is stable in the Tg2576 mouse, and MF BDNF may remain unchanged due to increased GC neuronal activity, since BDNF expression is well known to be activity dependent. The resistance of GCs to long-term Aβ accumulation provides an opportunity to understand how to protect vulnerable neurons from increased Aβ levels and therefore has translational implications.

## Significance Statement

Declining hippocampal brain-derived neurotrophic factor (BDNF) has been implicated in the pathogenesis of Alzheimer’s disease (AD). However, few studies have examined where hippocampal BDNF protein has its highest concentration, the dentate gyrus granule cell (GC) axons (mossy fibers; MFs). Using the well-established Tg2576 mouse model of AD, we found that MF BDNF did not decline with age, suggesting a notable exception to the idea that reduced hippocampal BDNF contributes to AD pathobiology. We also identified that Tg2576 GC activity correlates with MF BDNF protein, consistent with the activity dependence of BDNF. In addition, Tg2576 GCs were relatively resistant to accumulation of amyloid-β, providing insight into AD resilience, which has significant therapeutic implications.

## Introduction

Brain-derived neurotrophic factor (BDNF) is a member of the neurotrophin family of growth factors (Barde et al., 1982; Chao et al., 1998; Teng and Hempstead, 2004). BDNF is important in neuronal development and is also critical in the adult brain, where it supports neuronal structure and plasticity (Pardon, 2010; Park and Poo, 2013). One critical brain region where BDNF is high and has been studied extensively is the hippocampus, where it is considered to contribute to learning and memory (Yamada and Nabeshima, 2004; Minichiello, 2009).

AD brain tissues show variable BDNF levels, with some increases and some decreases depending on the brain area and the cell type being investigated. In hippocampus there is not always a significant change in AD (Ferrer et al., 1999; Holsinger et al., 2000; Garzon et al., 2002; Michalski et al., 2015), although some studies have reported that BDNF protein and mRNA decline (Phillips et al., 1991; Hock et al., 2000), and data from CA1 pyramidal neurons show a robust decline across the progression of dementia that correlates with cognitive decline and neuropathology (Ginsberg et al., 2006, 2010, 2019; Mufson et al., 2007; Nagahara et al., 2009). In animal models of AD, there are both increases and decreases in hippocampal BDNF mRNA and protein levels (Burbach et al., 2004; Szapacs et al., 2004; Peng et al., 2009). Despite the often equivocal findings, many investigators conclude that reduced BDNF occurs in AD, and contributes to it (Peng et al., 2009; Xue et al., 2022).

There are numerous studies of serum BDNF in AD, but serum levels may not be directly related to brain levels (Naegelin et al., 2018). One reason is that BDNF is expressed at high concentrations in platelets (Chacon-Fernandez et al., 2016). Nevertheless, reduced serum BDNF has been commonly reported in AD (Altar et al., 2009; Erickson et al., 2010; Allen et al., 2011; Thompson Ray et al., 2011; Autry and Monteggia, 2012; Tanila, 2017). Variability may be explained by the stage of AD, because two studies showed elevated serum BDNF early in AD, at the stage of mild cognitive impairment (MCI; Laske et al., 2007; Angelucci et al., 2010) followed by a decline (Laske et al., 2007). However, others found decreased serum BDNF both in MCI and later (Yu et al., 2008; Forlenza et al., 2010).

In normal rats and mice, BDNF protein shows abundant expression in the hippocampus in the mossy fiber (MF) axons of the dentate gyrus (DG) granule cells (GCs; Conner et al., 1997; Yan et al., 1997; Dieni et al., 2012). Despite the high expression in MFs, to our knowledge only one study has examined MF BDNF in AD and that study used patient-derived tissue (Connor et al., 1997). The results suggested a decreased MF BDNF protein in AD, but there was variation in age of the patients, drug history, postmortem delay, and other factors that limited conclusions.

In the present study, we took advantage of an antibody to BDNF that shows excellent specificity and staining for MF BDNF levels (Kolbeck et al., 1999; Dieni et al., 2012). We used an established AD mouse model, Tg2576 mice, which is advantageous because there is a slow development of amyloid-β (Aβ) plaques, occurring after 6 months of age (Citron et al., 1992; Hsiao et al., 1996; Kawarabayashi et al., 2001; Jacobsen et al., 2006).Therefore, we could reliably sample early (pre-plaque, 2–3 months old) or late (post-plaque, >11 months old) stages.

The results demonstrated that BDNF protein expression was strong in the MFs in Tg2576 mice and there was no detectable age-related decline. We then asked if the reason BDNF expression remains strong over the lifespan could be related to GC neuronal activity, because BDNF expression increases with activity (Tongiorgi et al., 2000) and many AD patients and mouse models of AD exhibit increased excitability (Palop and Mucke, 2010; Chin and Scharfman, 2013; Vossel et al., 2017). In this regard the Tg2576 mouse was useful because Tg2576 mice exhibit increased excitability *in vivo* (Bezzina et al., 2015; Kam et al., 2016) and in GCs *in vitro* (Alcantara-Gonzalez et al., 2021). We found that there were high levels of the transcription factor ΔFosB within GCs in Tg2576 mice when MF BDNF expression was relatively high, supporting the idea that increased GC activity promotes BDNF activity-dependent expression and could explain MF BDNF stability.

We then asked if stable GC BDNF expression might confer protection of GCs from Aβ deposition. Indeed, GCs showed remarkably little evidence of Aβ accumulation using several Aβ antibodies, even at 20 months of age. However, adjacent hilar neurons exhibited robust Aβ accumulation, as did hippocampal pyramidal cells. In summary, these data show BDNF protein in GC MFs is stable with age in Tg2576 mice, that there is a relationship to neuronal activity and the relative resistance of GCs to Aβ accumulation.

## Materials and Methods

### Experimental design

The study used the Tg2576 AD mouse model, with comparisons to wild type (WT) littermates. Two ages were chosen. The first age was 2–3 months, an age when mice are adult but have no sign of extracellular plaques. In addition, mice were selected from ages 11–20 months, when plaques are robust. At each age, both sexes and genotypes were included. In some experiments, the estrous cycle stage in female mice was estimated by sampling vaginal cytology at the time of death.

Before perfusion, mice were acclimated for at least 24 h to the laboratory where perfusion would occur. After perfusion, the brain was sectioned and processed using immunohistochemistry to evaluate BDNF protein expression, DG neuronal activity and Aβ immunoreactivity, as described below.

### Animal care and use

The experimental procedures were performed according to the National Institutes of Health guidelines and approved by the Institutional Animal Care and Use Committee at the Nathan Kline Institute. Mice were housed in standard mouse cages, with a 12 h light/dark cycle. Mice had food (Rodent diet 5001; LabDiet) and water *ad libitum*. During gestation and until weaning, mice were fed chow formulated for breeding (Formulab diet 5008; LabDiet). Mice were weaned at 23–25 d of age and then were fed a standard rodent chow after weaning (Rodent diet 5001, LabDiet). After weaning mice were housed with littermates of the same sex (maximum four mice per cage). For the experimental procedures, two ages were selected: 2–3 months (mean 98.5 ± 5.4 d; range 70–101, *n* = 12) and 11–20 months (mean 451.8 ± 23 d; range 337–611, *n* = 20). Ages in the Results that are in months were calculated by dividing the age in days by 30.3 because the average number of days/month is 30.3.

### Breeding and genotyping

Tg2576 mice express human APP695 with the Swedish (Lys670Arg, Met671Leu) mutations driven by the hamster prion protein promoter (Hsiao et al., 1996). They were bred in-house from male heterozygous Tg2576 and female nontransgenic mice (C57BL6/SJL F1 hybrid, Stock# 100,012, Jackson Labs). The genotype was determined using an in-house protocol for detecting the APP695 gene.

### Vaginal cytology

All mice were euthanized between 10:00 A.M. and 12:00 P.M. Cycle stage was estimated by assessment of vaginal cytology collected at the time of death. The characterization of the vaginal cells was based on Scharfman et al. (Scharfman et al., 2008). The cell types were defined as follows: leukocytes (round, small cells), epithelial cells (oval, intermediate-size, nucleated cells), and cornified epithelial cells (multipolar, large, nucleated cells; see Fig. 6). In young mice there was a pattern of vaginal cytology consistent with a cyclic pattern (D’Amour et al., 2015) and in old animals there was a pattern consistent with a cessation of cyclic estrous cycles, showing either a predominance of leukocytes or epithelial/cornified epithelial cells, suggesting a state of persistent diestrus or persistent estrus, respectively (Scharfman et al., 2008, 2009). In some mice, there were few cells in the vaginal sample each day. These animals were either old, and had entered reproductive senescence, or were young, and their estrous cycles may not have become regular yet. It is also possible that mice were not cycling well due to stress or other factors such as not being housed with cages of males nearby (Whitten, 1956; Scharfman et al., 2009; D’Amour et al., 2015). To quantify cell type and number, the number of visible cells in a field of view of 400 × 400 µm was manually counted using ImageJ (Schindelin et al., 2012).

### Anatomy

#### Perfusion-fixation and sectioning

Mice were deeply anesthetized by isoflurane inhalation (NDC#07-893-1389, Patterson Veterinary) followed by urethane [(Cat#U2500, Sigma-Aldrich), 250 mg/kg; stock solution 250 mg/ml in 0.9% sodium chloride (NaCl; Cat#S9888); intraperitoneal (i.p.)]. The abdominal cavity was opened with surgical scissors, followed by the heart cavity. A 26-gauge needle was inserted into the heart, followed by perfusion with 10 ml saline (0.9% NaCl in double distilled H_2_O; ddH_2_O) using a peristaltic pump (Minipuls 1; Gilson) followed by 30 ml of cold (4°C) 4% paraformaldehyde (PFA; Cat#19210, Electron Microscopy Sciences) in 0.1 M phosphate buffer (PB; note all buffers were pH 7.4). The brains were removed immediately and postfixed in 4% PFA at 4°C. Notably, when tissue was postfixed with 4% PFA overnight, BDNF antibodies performed poorly. Therefore, shorter durations of postfixation in 4% PFA were tested (1, 2, and 3 h). Three hours of postfixation was chosen because it optimized BDNF staining by reducing PFA exposure and maintained tissue integrity during processing better than shorter postfixation times. In addition, tissue integrity was improved by transferring sections with Pasteur pipettes that were heated to melt the tip into a curved shape, instead of brushes. Sections were transferred after floating them onto the curved part of the pipette.

#### Sectioning

After postfixation, the brains were washed in 0.1 M PB. Before sectioning, the brains were hemisected. One hemisphere was cut in the coronal plane and the other in the horizontal plane (50-µm-thick sections) using a vibratome; (Model# VT1000p, Leica). Similar septotemporal levels were selected and processed together. The sections were 300 µm apart. The sections were collected in 0.1 M PB. Sections that were not used immediately were stored at −20°C in 30% sucrose (Cat#S8501, Sigma-Aldrich) and 30% ethylene glycol (Cat#293237, Sigma-Aldrich) diluted in 0.1 M PB. We detected no difference in sections stored in 0.1 M PB and the storage solution.

#### Immunohistochemistry

##### BDNF immunostaining: fluorescence and brightfield

BDNF protein was detected with a mouse monoclonal anti-BDNF antibody that has been validated [(Mab#9, Developmental Hybridoma Bank] using either a fluorescence or brightfield protocol. This antibody is similar qualitatively to the one that was used originally in normal rodents that demonstrated robust MF staining (Conner et al., 1997) and was characterized and validated as a mossy fiber marker (Dieni et al., 2012).

For immunofluorescence, free-floating sections were first washed in 0.1 M Tris buffered saline (TBS, three washes for 5 min each) and then blocked with 3% mouse-on-mouse blocking serum (M.O.M; Cat#MKB-2213, Vector Laboratories) in TBS for 1 h. The primary antibody was diluted in a solution of 3% bovine serum albumin (BSA; Cat#A7906, Sigma-Aldrich), 2% donkey serum (DS; D9663, Sigma-Aldrich), and 0.3% Triton X-100 (Cat#X-100, Sigma-Aldrich) in TBS to yield a final concentration of 10 µg/ml anti-BDNF. Sections were incubated for 2 nights at 4°C on a rotator (The Belly Dancer, Stovall). For detection, donkey anti-mouse IgG-488 Alexa Fluor–conjugated secondary antibody was used (1:500; Cat#A21202, Invitrogen) in a solution of 2% DS and 0.3% Triton X-100 in TBS. Sections were incubated for 1 h at room temperature (RT), washed in TBS, and then TB. Washes were three for 5 min each. Labeled sections were mounted onto glass slides and coverslipped with fluorescent mounting medium (Citofluor AF1; Cat#17970-25, Electron Microscopy Sciences).

For brightfield microscopy, free-floating sections were first washed in TBS (3 washes for 5 min each) and then treated with 0.25% H_2_O_2_ (Cat#216763, Sigma-Aldrich), in TBS for 3min. After three washes of 5 min each in TBS, sections were incubated in 0.3% Triton X-100 in TBS for 20 min at RT. Next, sections were blocked in 1% BSA, 5% normal horse serum (NHS; Cat#S-2000, Vector Laboratories), and 1.5% M.O.M in TBS for 1 h at RT. The primary antibody was diluted in a solution of 1% BSA, 5% NHS, and 0.3% Triton X-100 in TBS to yield a final concentration of 10 µg/ml anti-BDNF. Sections were incubated for 2 nights at 4°C on a rotator. Then sections were incubated in biotinylated horse anti-mouse IgG secondary antibody (1:500; Cat#BA-2000, Vector Laboratories) in 1% BSA, and 0.3% Triton X-100 in TBS for 3 h at RT, followed by Avidin-Biotin-Complex (ABC; ABC Elite kit; 1:1,000; Cat#PK-6100, Vector Laboratories) in 1% BSA in TBS for 1 h at RT. Sections were rinsed in TBS and then in Tris buffer (TB). There were 3 washes for 5 min each. Next, sections were reacted with 3,3′-diaminobenzidine (DAB; Cat#DS905, Sigma-Aldrich; 50 mg/100 ml in 0.1 M TB) in 40 µg/ml ammonium chloride (NH_4_Cl; Cat#A4514, Sigma-Aldrich), 2 mg/ml D(+)–glucose (Cat#G5767, Sigma-Aldrich), 10 mM nickel chloride (NiCl_2_; Cat#N6136, Sigma-Aldrich), 3 µg/ml glucose oxidase (Cat#G2133-50KU, Sigma-Aldrich), and then washed (3 times for 5 min each) in TB. Labeled sections were mounted on gelatin-coated slides (1% bovine gelatin; Cat#G9391, Sigma-Aldrich) and dried overnight at RT. On the next day, sections were dehydrated with increasing concentrations of ethanol (70%, 2.5 min; 95%, 2.5 min; 100%, 5 min), cleared in Xylene (4 min; Cat#534056, Sigma-Aldrich), and coverslipped with Permount (Cat#17986-01; Electron Microscopy Sciences).

Sections were examined using an upright microscope (BX61, Olympus) equipped with brightfield and fluorescence detection. Sections were photographed using a digital camera (Model RET 2000R-F-CLR-12, Q-Imaging) and acquired using ImagePro Plus, v.7.0 (Media Cybernetics). For both fluorescence and brightfield protocols, sections from WT and Tg2576 were processed and photographed together, using the same microscope and software settings. Figures were composed in Photoshop (v7.0, Adobe).

##### ΔFosB

Sections from WT and Tg2576 were postfixed for 1 h in 4% PFA. Free-floating sections were first washed in 0.1 M TB (3 washes for 5 min each) and treated with 1% H_2_O_2_ in TB for 3 min. After 3 washes in TB (5 min each), sections were incubated in 0.25% Triton X-100 in TB (TrisA) and subsequently in 1% BSA and 0.25% Triton X-100 in TB (TrisB), for 10 min each. Then sections were blocked in 10% normal goat serum (NGS; Cat#S-1000, Vector Laboratories), 1% BSA and 0.25% Triton X-100 in TB for 1 h at RT. The primary antibody, a rabbit monoclonal anti-ΔFosB antibody (Cat#D358R, Cell Signaling), was diluted in TrisB (final concentration, 1:1,000). Sections were incubated overnight at 4°C on a rotator. On the following day, sections were rinsed in TrisA and subsequently in TrisB for 10 min each, then incubated in biotinylated goat anti-rabbit IgG secondary antibody (1:500; Cat#BA-1000, Vector Laboratories), diluted in TrisB, for 60 min at RT, followed by 2 rinses of 10 min each in TrisA, then TrisB. Next, sections were incubated in ABC (ABC Elite kit; 1:1,000) and diluted in TrisB for 2 h at RT. Sections were rinsed 3 times in TB (5 min each) reacted with DAB (50 mg/100 ml in 0.1 M TB), and then mounted and coverslipped as for BDNF (described above). Sections were examined and photographed, and figures were prepared as for BDNF.

##### Aβ immunostaining

Aβ-immunofluorescence (Aβ-IF) was detected primarily with an antibody to human Aβ (McSA1) which was raised against the N-terminal fragment (residues 1–12; Grant et al., 2000). This antibody can detect the soluble and insoluble forms of Aβ, with a specificity for Aβ without labeling amyloid precursor protein (APP, Grant et al., 2000; Billings et al., 2005). We adapted a protocol from Kobro-Flatmoen et al. (2016) using free-floating sections. First, sections were postfixed in 4% PFA to improve tissue integrity during the antigen retrieval procedure. After 3 h of postfixation, sections were washed in 0.1 M PB (3 washes for 5 min each) and then treated for 3 h in 0.1 M PB (60°C). All the following washes and dilutions were performed in 0.1 M PB. Sections were incubated for 20 min in 0.5% Triton X-100. Sections were then blocked for 2 h (5% NGS) and incubated overnight on a rotator at 4°C in primary antiserum (1:1,000, mouse monoclonal antibody to Aβ; Cat#MM-015-P, MédiMabs), 3% NGS, and 0.5% Triton X-100.

Two validated, conventionally-used Aβ antibodies (Li et al., 2017; Perez et al., 2019) were used to confirm results with McSA1: a mouse monoclonal antibody to Aβ residues 1–16 (1:1,000; clone 6E10; Cat#803001, Biolegend) or a mouse monoclonal antibody to Aβ residues 17–24 (1:1,000; clone 4G8; Cat#800708, Biolegend). For all Aβ antibodies, incubation with primary antisera was followed by 2 h of incubation with secondary antibody (1:350, goat anti-mouse IgG Alexa Fluor 488; Cat#A1101, Invitrogen).

Sections were examined using a fluorescence microscope (BX61, Olympus) and photographed using a digital camera (Model# Infinity 3-6UR, Lumenera) and the Infinity 3 software (v. 6.5.6, Lumenara). As for other photography described above, sections from WT and Tg2576 were processed and photographed together, using the same microscope and software settings.

#### Thioflavin-S staining

Aβ plaques were detected following the protocol of Roberson et al. (2007). Sections from WT and Tg2576 were mounted on 0.1% gelatin-coated slides, incubated in a thioflavin-S solution (1% thioflavin-S, Cat#T1892, Sigma-Aldrich, in ddH_2_O) for 10 min at RT, dehydrated in a graded series of ethanol (80%, 4 min; 95%, 4 min; 100%, 4 min), cleared in Xylene (4 min), and coverslipped as for BDNF (described above). Sections were examined and photographed as described above for BDNF.

#### Quantification

##### BDNF

There were two methods used for quantification.

- Quantification of BDNF-immunoreactivity (BDNF-ir) was done as before (Skucas et al., 2013) by first defining three regions of interest (ROIs) in the stratum lucidum (SL), the location of the BDNF-rich MF projections. These three ROIs were at the end of the MF plexus where BDNF-ir is highest. The background value was measured from an ROI in SR, where BDNF-ir is relatively weak. BDNF-ir was calculated by subtracting the background value for optical density (OD) from the average OD of the three ROIs in SL. Then, MF BNDF-ir was normalized to the background of the same section. To quantify the BDNF-ir in the dorsal hippocampus, sections were chosen in the region ranging from the septal pole to the point in the rostral–caudal axis where the hippocampus begins to descend ventrally (1.34–2.18 mm caudal distance to Bregma). Ventral sections were chosen where the outline of the DG assumes a C-shape (2.30–3.16 mm caudal distance to Bregma).
- A second method of quantification examined the entire MF projection from the GCL to CA2. The background value was measured as described above. BDNF-ir was calculated by subtracting the background value from the value of the ROI of the MFs. MF BDNF-ir quantification was then done as described above.

##### ΔFosB

Quantification of ΔFosB-immunoreactivity (ΔFosB-ir) was done by first defining an ROI that encircled the upper blade of the GC layer (GCL). The GCL was defined as the location of packed GC somata. The borders with the hilus and inner molecular layer were defined as the location where GCs were no longer adjacent to one another, i.e., they were >1 GC cell body width apart, as previously described (Bermudez-Hernandez et al., 2017).

The level of ΔFosB immunoreactivity (ΔFosB-ir) in the GCL was quantified using ImageJ software (NIH) at 40× magnification. Images were thresholded to create a binary overlay in which ΔFosB-positive nuclei were above threshold and the background was below threshold similar to other studies of GCs stained with an antibody to Prox1 (Bermudez-Hernandez et al., 2017) or other immunocytochemical studies of c-Fos-ir GCs (Duffy et al., 2013; Moretto et al., 2017). For a GC to be considered positive, its ΔFosB-ir had to be >2× the background. Three dorsal sections were used to calculate a mean for a given mouse.

ΔFosB-ir in GCs was quantified by manually counting ΔFosB-positive cell bodies in the upper blade of DG GCL. The same threshold used to quantify ΔFosB-ir in the GCL was used to define a ΔFosB-ir positive GC.

##### McSA1, 6E10, and 4G8

###### CA1

Quantification of Aβ-IF was done by first defining three ROIs within the area CA1 cell layer that included the highest levels of fluorescence. The background value was measured by an ROI in SLM where IF was not detected. Aβ-IF was calculated by subtracting the background from the average of the values of intensity of the three ROIs. Aβ-IF for each section was then normalized [(optical density)—(background optical density]/(background optical density). This approach has also been used previously for other immunocytochemical studies (Lee et al., 2012; Skucas et al., 2013).

##### DG

###### GCs

Aβ-IF in GCs was quantified from an ROI encircling the upper blade of the GCL. The background value was measured by an ROI in SR, where the Aβ-IF is weak. Aβ-IF was calculated by subtracting the background from the average intensity of the ROI for the GCL. Aβ-IF for each section was then normalized, as described above.

###### Hilar cells

Aβ-IF in hilar cells was quantified by manually counting Aβ-IF cell bodies in the hilus of the DG. A positive hilar cell was defined by choosing a threshold value which was based on the value for a bright cell in the Tg2576 mice. The threshold had to be high enough so that WT hilar cells would not reach threshold. This choice of threshold was straightforward because immunofluorescence of Tg2576 hilar cells was so bright and immunofluorescence of WT hilar cells was so hard to detect (see figures). The hilus was defined as zone 4 of Amaral (1978).

To compare the immunofluorescence of CA1 and the hilus, the highest optical density measurement in the CA1 pyramidal cells was compared to the highest value for hilar cells.

#### Statistical analysis

All data are presented as the mean ± standard error of the mean (SEM). The statistical significance was set at *p* < 0.05. Statistical analyses were performed using GraphPad Prism Software (https://www.graphpad.com/scientific-software/prism/, RRID: SCR_002798).

Parametric tests were used when data fit a normal distribution, determined by the Shapiro–Wilk normality test, and variance was homogeneous, determined by the Brown–Forsythe test. An unpaired *t* test was used for two groups. A two-way ANOVA followed by Tukey’s multiple comparisons test were used when comparing two independent variables. Interactions of factors are not reported in the Results unless they were significant. For data that did not follow a normal distribution or showed significant heteroscedasticity, nonparametric tests were selected. The Mann–Whitney *U* test was used for two groups. A Kruskal Wallis test followed by Dunn’s test were used when two or three independent variables were compared.

## Results

### Modified procedures for BDNF immunohistochemistry

**Figure 1.**
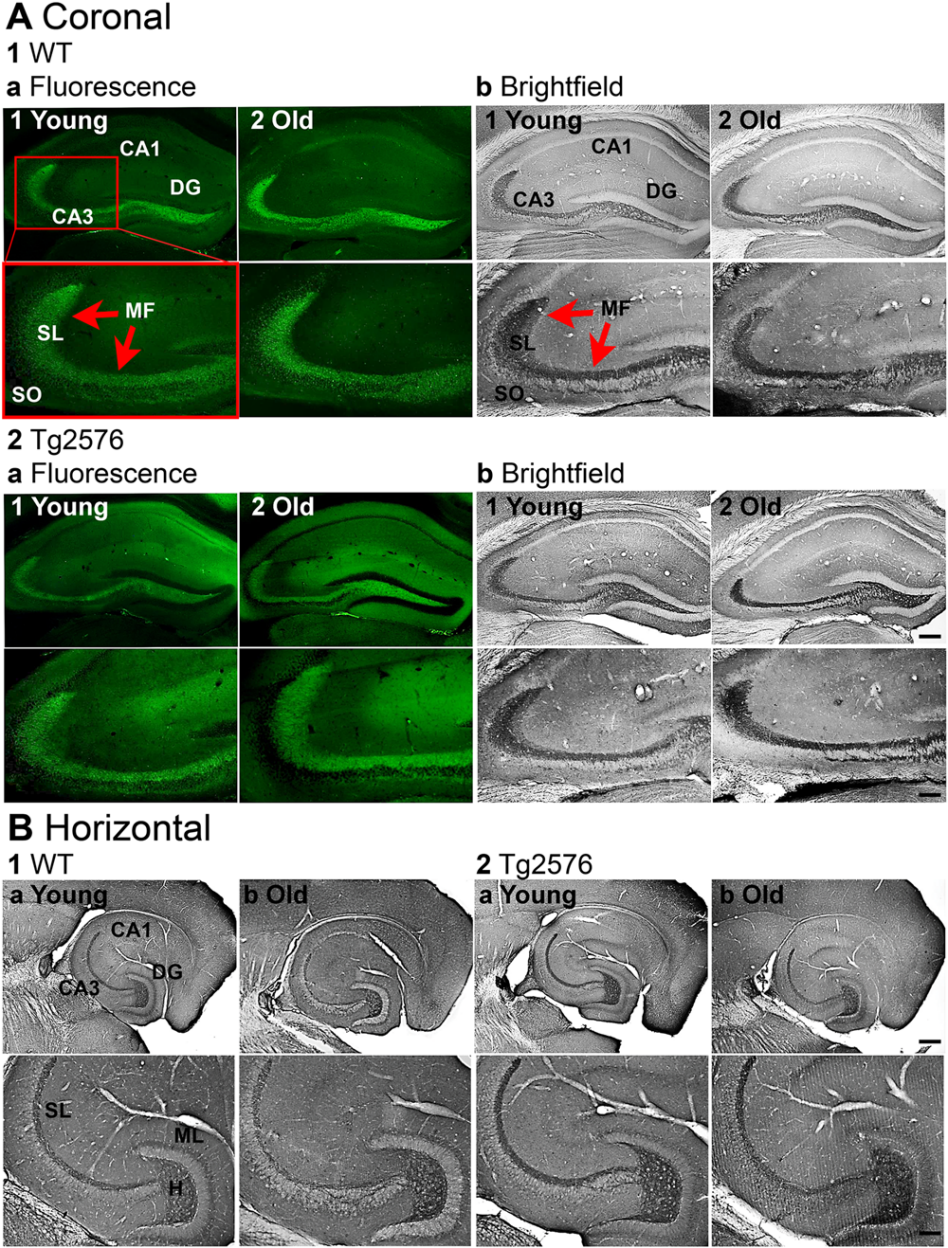
Modified methods allowed visualization of robust BDNF protein expression of in GC mossy fibers (MFs). ***A***, Representative examples of BDNF-immunoreactivity (BDNF-ir) in coronal sections of dorsal hippocampus. Calibration bar is in 2b2 and applies to all images. Top images, 200 µm; Bottom images, 100 µm. SL, stratum lucidum; SO, stratum oriens. 1. WT mice. Intense BDNF-ir was found in MFs (red arrows). a. Immunofluorescence in a young (1, 3.2 months-old) and old (2, 12.3 months-old) mouse. b. DAB-ir in a young (1, 3.3 months-old) and old (2, 11.1 months-old) mouse. 2. Tg2576 mice. a. Immunofluorescence in a young (1, 3.2 months-old) and old (2, 12.2 months-old) mouse. b. DAB-ir a young (1, 3.3 months-old) and old (2, 10.9 months-old) mouse. ***B***, Representative examples of BDNF-ir in horizontal sections of ventral hippocampus. 1. WT mice. BDNF-ir was detected in MFs in SL and the hilus (H). Calibration bar is in 2b2. Top, 200 µm; Bottom, 100 µm. ML, molecular layer. a-b. DAB-ir in a young (a, 3.3 months-old) and old (b, 14.2 months-old) mouse. 2. Tg2576 mice. a-b. DAB-ir in a young (a, 3.3 months-old) and old (b, 14.2 months-old) mouse.

We optimized an immunohistochemical protocol that allowed us to evaluate the expression of BDNF protein of WT and Tg2576 mice. This modification included a reduced exposure to PFA and instrumenting procedures to handle the weakly fixed, and therefore fragile, tissue sections. Hippocampal sections were incubated with a highly speciﬁc afﬁnity-puriﬁed monoclonal antibody to BDNF (see Materials and methods; Fig. 1). Two different protocols were used, one for immunofluorescence microscopy and the other for DAB brightfield illumination (Fig. 1). The results were similar for both protocols. BDNF protein expression was predominantly in the MFs (Fig. 1*A,B*). A similar pattern of expression was identified both in coronal (Fig. 1*A*) and horizontal sections (Fig. 1*B*).

### MF BDNF protein is not significantly different in young and old Tg2576 mice

**Figure 2.**
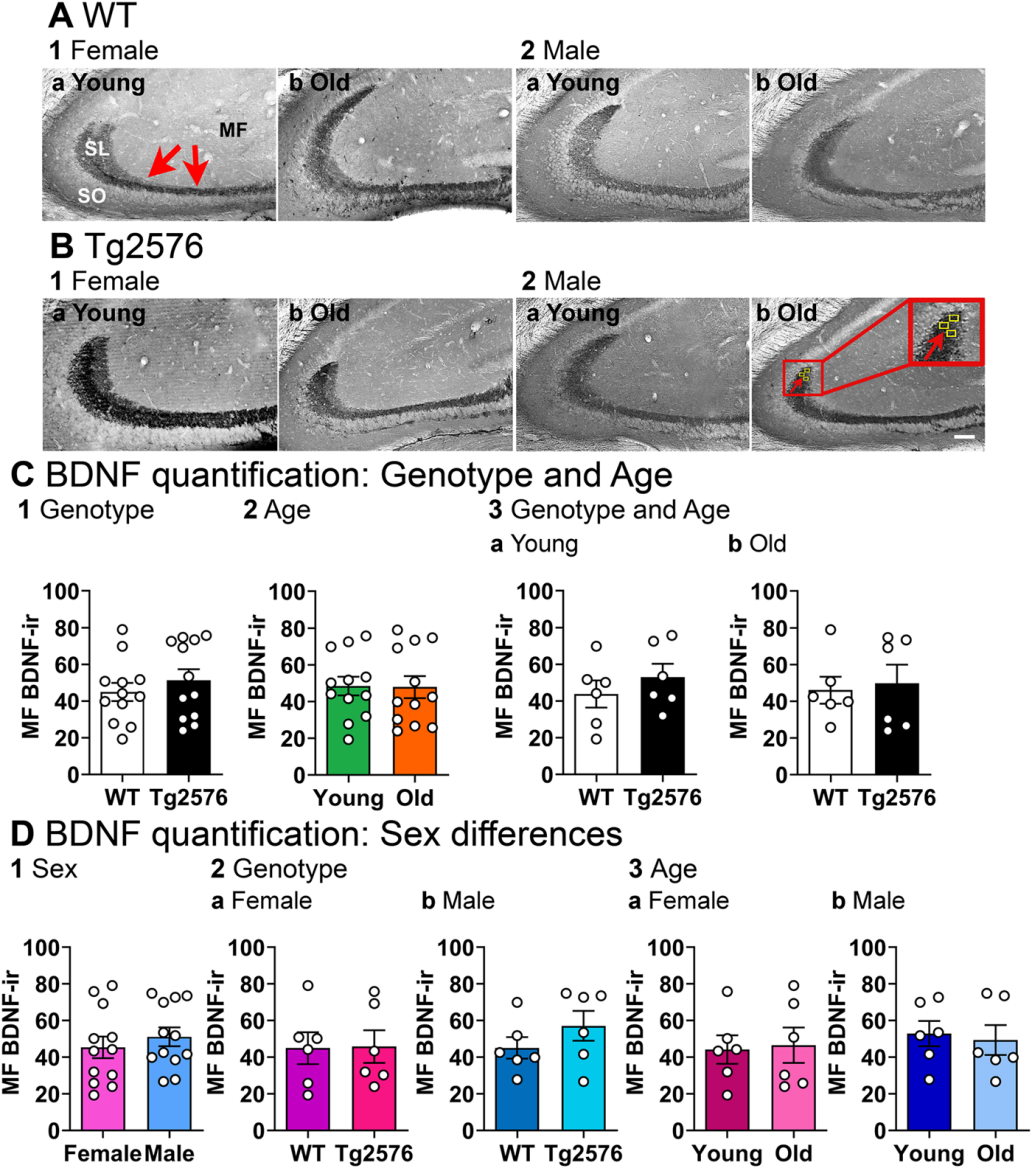
No detectable differences in BDNF protein with age and no sex differences. ***A***, WT mice. Representative examples of MF BDNF-ir (red arrows) in coronal sections of dorsal hippocampus. Calibration bar is in 2b and is 100 µm. SL, stratum lucidum; SO, stratum oriens, MF, mossy fibers. 1. Female mice. a, b. A section from a young (a, 2.5 months-old) and old (b, 14.2 months-old) mouse is shown. 2. Male mice. a-b. A section from a young (a, 3.2 months-old) and old (b, 12.3 months-old) mouse is shown. ***B***, Tg2576 mice. Representative examples are shown. Inset shows the area of quantification (yellow boxes marked by arrows). 1. Female mice. a-b. A section from a young (a, 3.5 months-old) and old (b, 14 months-old) mouse is shown. 2. Male mice. a, b. A section from a young (a, 3.3 months-old) and old (b, 12 months-old) mouse is shown. ***C***, 1. A Mann–Whitney *U* test was conducted to compare genotypes with sexes pooled. There was no significant difference in MF BDNF-ir (*p* = 0.514, *n* = 12/group). 2. To examine possible differences in ages, genotypes were pooled. There was no significant difference in MF BDNF-ir of young and old mice (unpaired *t* test, *p* = 0.943, *t* = 0.072, df = 22). 3. There was no effect of genotype on MF BDNF-ir at different ages. a. Young WT and young Tg2576 mice were not different (unpaired *t* test, *p* = 0.392, *t* = 0.089, df = 10). b. Old WT and old Tg2576 mice were not different (Mann–Whitney *U* test, *p* = 0.937, old WT vs old Tg2576 *n* = 6/group). ***D***, 1. An unpaired *t* test was conducted to compare sexes. Genotypes were pooled. There were no significant sex differences (*p* = 0.470, *t* = 0.735, df = 22). 2. There was no effect of genotype on MF BDNF-ir in different sexes. Ages were pooled. a. Female WT and female Tg2576 mice showed no differences (unpaired *t* test, *p* = 0.945, *t* = 0.070, df = 10). b. Male WT and male Tg2576 mice were not different (unpaired *t* test, *p* = 0.258, *t* = 1.199, df = 10). 3. There was no effect of age on MF BDNF-ir in different sexes. a. Young female and old female mice were not different (unpaired *t* test, *p* = 0.850, *t* = 0.194, df = 10). b. Young and old male mice were not different (unpaired *t* test, *p* = 0.747, *t* = 0.331, df = 10). See Table 2-1 for more details.

We tested the hypothesis that BDNF protein expression changed with age. For this goal we investigated BDNF-ir in Tg2576 mice and in their WT littermates at two different ages, 2–3 months, and >11 months. The early age of 2–3 months was chosen because it is considered to be an age early in the development of AD-like features such as the appearance of plaques (as explained in the Introduction). The older age of >11 months was selected because plaques have developed by that age. Initial studies of mice >11 months was limited to 11–16 months to keep the age range of the older group from being too broad. Representative examples of coronal sections from each experimental group are shown in Figure 2*A* and *B*. The groups were young WT, old WT, young Tg2576 and old Tg2576 (*n* = 6/group, 3 males and 3 females).

10.1523/ENEURO.0192-23.2023.t2-1 Extended Data Table 2-1 Normality and homogeneity of variance assessment for Figure 2, where MF BDNF-ir was quantified. A non-parametric test was used for the statistical analysis when either the data did not fit a normal distribution or there was significant heteroscedasticity of variance. Download Extended Data Table 2-1, DOC file.

First, we asked if there was a difference in WT and Tg2576 MF BDNF. There were no significant differences in MF BDNF-ir related to genotype when we pooled sexes (Mann–Whitney *U* test, *p* = 0.514, WT *n* = 12, Tg2576 *n* = 12; Fig. 2*C*1). There was no significant effect of age when we pooled genotypes (unpaired *t* test, *p* = 0.943, *t* = 0.072, df =22; Fig. 2*C*2). At young ages, WT and Tg2576 MF BDNF-ir were not significantly different (sexes pooled; unpaired *t* test, *p* = 0.392, *t* = 0.089, df = 10; Fig. 2*C*3*a*). MF BDNF protein in old Tg2576 mice was not significantly different from old WT mice (Mann–Whitney *U* test, *p* = 0.937, old WT *n* = 6, old Tg2576 *n* = 6; Fig. 2*C*3*b*). These data suggest that MF BDNF-ir did not decline with age.

In Figure 2*D*, we focused on potential sex differences with additional analyses. There were no significant effects of sex on MF BDNF protein (genotypes pooled, unpaired *t* test, *p* = 0.470, *t* = 0.735, df = 22; Fig. 2*D*1). WT and Tg2576 females were not significantly different (unpaired *t* test, *p* = 0.945, *t* = 0.070, df = 10, Fig. 2*D*2*a*), and WT and Tg2576 males were not significantly different either (unpaired *t* test, *p* = 0.258, *t* = 1.199, df = 10; Fig. 2*D*2*b*). There also did not appear to be a decline in MF BDNF-ir with age in females, because there were no significant differences in young and old females (unpaired *t* test, *p* = 0.850, *t* = 0.194, df = 10; Fig. 2*D*3*a*) or young and old males (unpaired *t* test, *p* = 0.747, *t* = 0.331, df = 10; Fig. 2*D*3*b*). The results of tests for normality and homogeneity of variance are shown in Extended Data Table 2-1.

**Figure 3.**
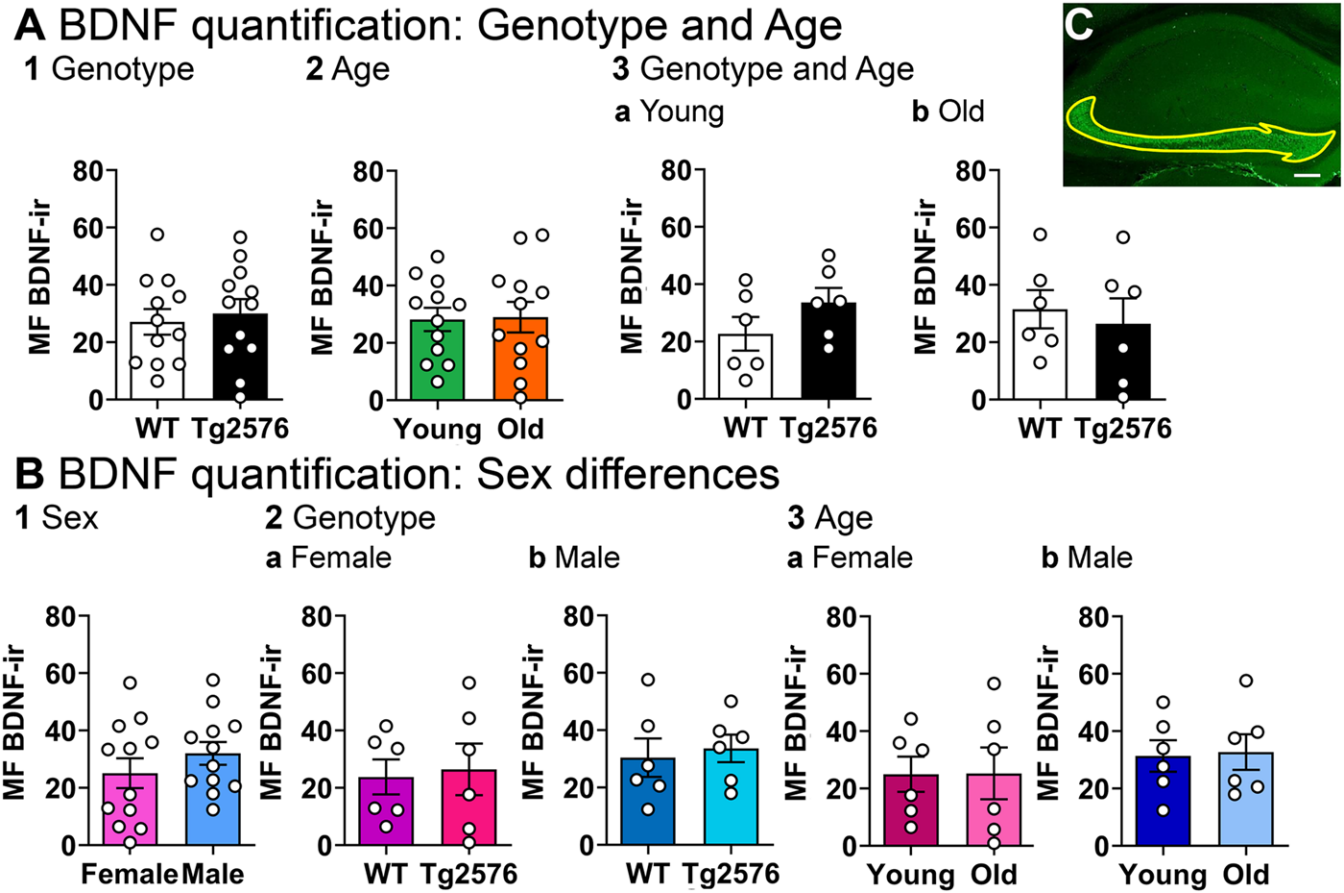
Confirmation of results using a second method of MF BDNF protein quantification. ***A***, 1. An unpaired *t* test was conducted to compare genotypes with sexes pooled. There was no significant difference in MF BDNF-ir (*p* = 0.666, *t* = 0.438, df = 22). 2. To examine possible differences in ages, genotypes were pooled. There was no significant difference in MF BDNF-ir of young and old mice (unpaired *t* test, *p* = 0.906, *t* = 0.120, df = 22). 3. There was no effect of genotype on MF BDNF-ir at different ages. a. Young WT and young Tg2576 mice were not different (unpaired *t* test, *p* = 0.188, *t* = 1.412, df = 10). b. Old WT and old Tg2576 mice were not different (unpaired *t* test, *p* = 0.656, *t* = 0.458, df = 10). ***B***, 1. An unpaired *t* test was conducted to compare sexes. Genotypes were pooled. There were no significant sex differences (*p* = 0.303, *t* = 1.055, df = 22). 2. There was no effect of genotype on MF BDNF-ir in different sexes. Ages were pooled. a. Female WT and female Tg2576 mice showed no differences (unpaired *t* test, *p* = 0.813, *t* = 0.242, df = 10). b. Male WT and male Tg2576 mice were not different (unpaired *t* test, *p* = 0.706, *t* = 0.389, df = 10). 3. There was no effect of age on MF BDNF-ir in different sexes. a. Young female and old female mice were not different (unpaired *t* test, *p* = 0.982, *t* = 0.022, df = 10). b. Young and old male mice were not different (unpaired *t* test, *p* = 0.873, *t* = 0.164, df = 10). ***C***, Representative example of MF BDNF-ir quantification (yellow ROI) in coronal sections of dorsal hippocampus. Calibration bar is 200 µm. See Table 3-1 for more details.

These data were confirmed with a second method to quantify BDNF-ir. We quantified the BDNF-ir in coronal sections, measuring the entire MF projection from the GCL to CA2 (Fig. 3*C*). There were no significant differences in MF BDNF-ir related to genotype when we pooled sexes (unpaired *t* test, *p* = 0.666, *t* = 0.438, df = 22; Fig. 3*A*1). There was no significant effect of age when we pooled genotypes (unpaired *t* test, *p* = 0.906, *t* = 0.120, df = 22; Fig. 3*A*2). At young ages, WT and Tg2576 MF BDNF-ir were not significantly different (sexes pooled; unpaired *t* test, *p* = 0.188, *t* = 1.412, df = 10; Fig. 3*A*3*a*). MF BDNF protein in old Tg2576 mice was not significantly different from old WT mice (unpaired *t* test *t* test , *p* = 0.656, *t*= 0.458, df = 10; Fig. 3*A*3*b*).

10.1523/ENEURO.0192-23.2023.t3-1 Extended Data Table 3-1 Normality and homogeneity of variance assessment for Figure 3. Which also shows quantified MF BDNF-ir. Download Extended Data Table 3-1, DOC file.

When we focused on sex (Fig. 3*B*), the results did not show differences either. Thus, there were no significant effects of sex on MF BDNF protein (genotypes pooled, unpaired *t* test, *p* = 0.303, *t* = 1.055, df = 22; Fig. 3*B*1). WT and Tg2576 females were not significantly different (unpaired *t* test, *p* = 0.813, *t* = 0.242, df = 10, Fig. 3*B*2*a*), and WT and Tg2576 males were not significantly different either (unpaired *t* test, *p* = 0.706, *t* = 0.389, df = 10; Fig. 3*B*2*b*). There also were no significant differences in young and old females (unpaired *t* test, *p* = 0.982, *t* = 0.022, df = 10; Fig. 3*B*3*a*) or young and old males (unpaired *t* test, *p* = 0.873, *t* = 0.164, df = 10; Fig. 3*B*3*b*). The results of tests for normality and homogeneity of variance are shown in Extended Data Table 3-1.

**Figure 4.**
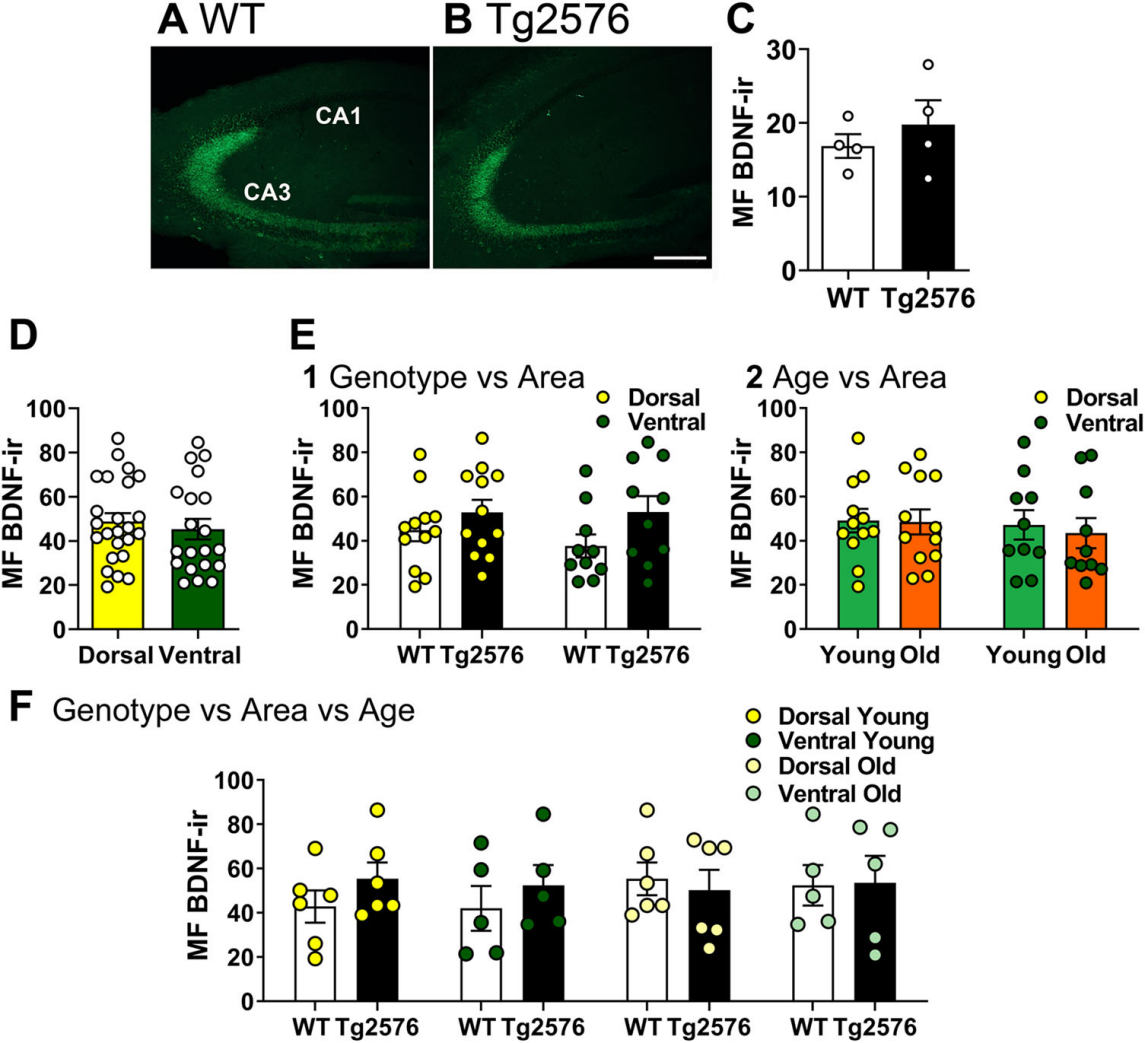
Confirmation that there were no detectable differences in BDNF protein at very old ages and across the septotemporal axis. ***A,B***, Representative examples of MF BDNF-IF in coronal sections of dorsal hippocampus of WT and Tg2576mice. Calibration bar is (in ***B***) and is 100 µm. ***C***, An unpaired *t* test was conducted to compare genotypes with sexes pooled. There was no significant difference in MF BDNF-IF (*p* = 0.457, *t* = 0.795, df = 6). ***D***, A Mann–Whitney *U* test was conducted to compare dorsal and ventral regions. Genotypes were pooled. There were no significant sex differences (*p* = 0.514, dorsal *n* = 24, ventral *n* = 20). ***E***, 1. There was no significant effect of genotype or area (dorsal or ventral) when ages were pooled (two-way ANOVA, genotype: (*F*_(1,40)_ = 4.047, *p* = 0.051; area: *F*_(1,40)_ = 0.036, *p* = 0.553). 2. There was also no significant effect of age or area (dorsal or ventral) when genotypes were pooled (Kruskal-Wallis test, *H* = 0.908, *p* = 0.8234, dorsal). ***F***, There was no significant effect of genotype, area or age on MF BDNF protein expression (Kruskal-Wallis test, *H* = 4.921, *p* = 0.670). See Table 4-1 for more details.

We then asked if there would be a decline in MF BDNF-ir if older mice were used. Therefore, we used 17- to 20-month-old mice (Fig. 4*A,B*). We confirmed the results at younger ages, that MF BDNF protein was similar in WT and Tg2576 mice (unpaired *t* test, *p* = 0.457, *t* = 0.795, df = 6, Fig. 4*C*).

10.1523/ENEURO.0192-23.2023.t4-1 Extended Data Table 4-1 Normality and homogeneity of variance assessment for Figure 4, where >17 month-old mice and ventral MF BDNF-ir was quantified. Note that for a three-way ANOVA (F in the Table), normality evaluation was not possible because the n was 3/group. Therefore, a non-parametric test was used for statistical comparisons. Download Extended Data Table 4-1, DOC file.

**Figure 5.**
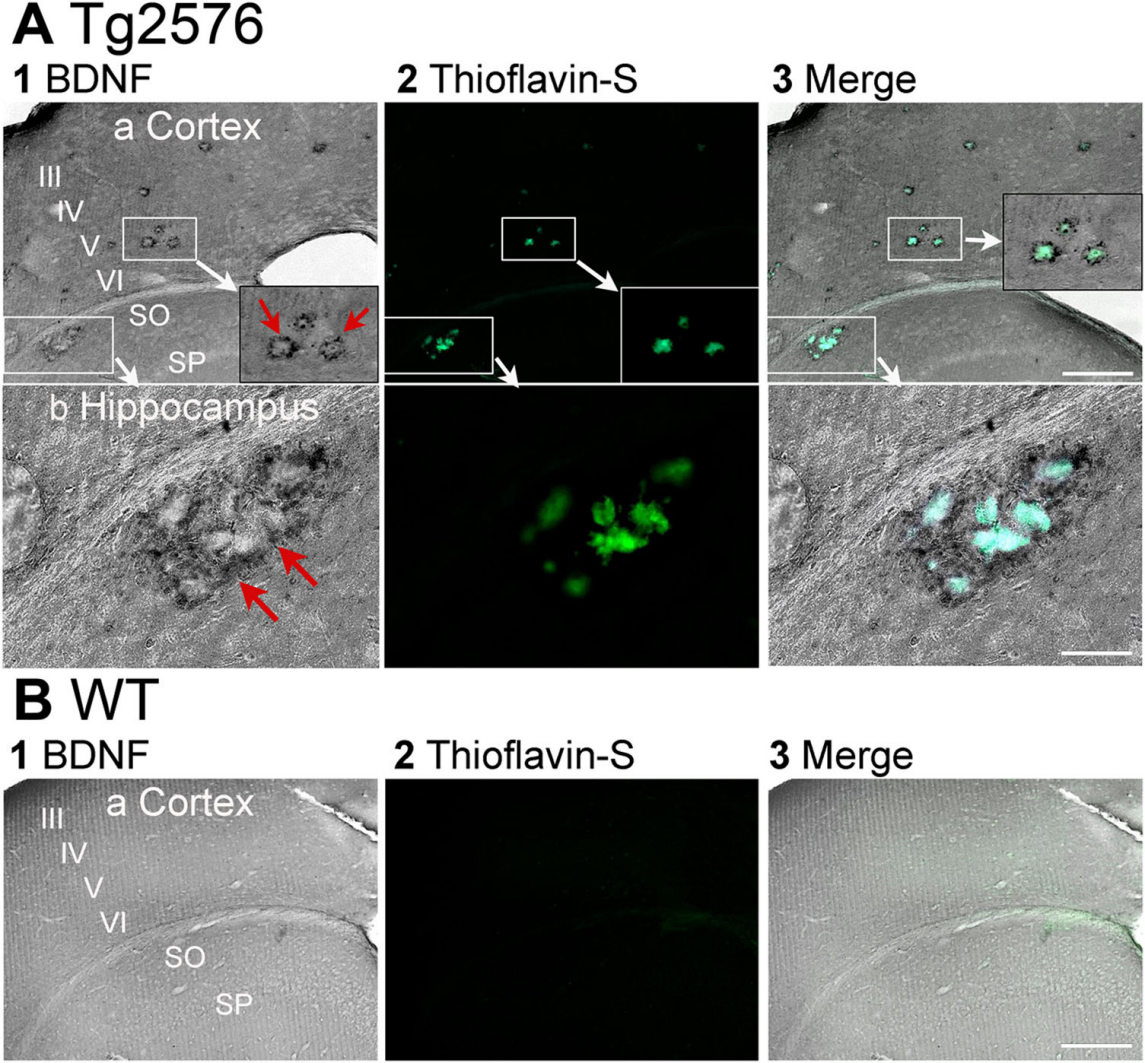
BDNF protein is present around hippocampal and cortical plaques in Tg2576 mice. ***A***, 1. BDNF-ir in a section from a Tg2576 mouse (14.2 months-old) shows BDNF-ir surrounding what appear to be extracellular plaques. Calibration bar is in 3. a, 200 µm. b, 100 µm. SO, stratum oriens. SP, stratum pyramidale. a. Cortex. The cortex above hippocampus is shown. Layers III-VI are marked. There are two boxes that surround extracellular plaques. One box in cortex is expanded at the lower right as marked by the white arrow. It shows BDNF-ir surrounding what appear to be plaques (red arrows). b. Hippocampus. The second box in A1 is expanded to show the BDNF-ir around plaques in area CA1. 2. The same section was stained for thioflavin-S after immunocytochemistry using the antibody to BDNF. It shows the areas of BDNF-ir are around thioflavin-S staining. 3. A merged image shows the thioflavin-S staining and BDNF-ir overlap. Note that DAB-ir was dark grey and thioflavin-S staining was green, and the merge showed a blue color where there was double-staining. ***B***, 1-3. BDNF-ir in a section from a WT mouse (17.2 months-old) shows no presence of plaques stained for thioflavin-S surrounded by BDNF-ir in cortex. Calibration bar is in 3, 200 µm.

In Figure 4*D–F*, we focused on potential differences in MF BDNF protein expression in hippocampus across the septotemporal axis. Dorsal and ventral MF BDNF-ir were not significantly different when genotypes were pooled (Mann–Whitney *U* test, *p* = 0.514, dorsal *n* = 24, ventral *n* = 20, Fig. 4*D*). We also did additional statistics to further analyze dorsal and ventral regions. When a two-way ANOVA was conducted with genotype and area (dorsal vs ventral) as factors, there was no significant effect of genotype (*F*_(1,40)_ = 4.047, *p* = 0.051) or area (*F*_(1,40)_ = 0.036, *p* = 0.553; Fig. 4*E*1). Similarly, no difference was found when age and area (dorsal vs ventral) were compared (Kruskal–Wallis test, age: *H* = 0.908, *p* = 0.823, dorsal *n* = 12/group, ventral *n* = 10/group; Fig. 4*E*2). A Kruskal–Wallis test comparing the genotype, area, and age confirmed the lack of effects (*H* = 4.921, *p* = 0.670, dorsal *n* = 6/group, ventral *n* = 5/group; Fig. 4*F*). The results of tests for normality and homogeneity of variance are shown in Extended Data Table 4-1. Although these results suggested that there were no detectable abnormalities in the level of MF BDNF protein in Tg2576 mice compared to WT, there could have been abnormalities of another kind or in another brain area. Indeed, we found BDNF-ir in and around extracellular plaques both in hippocampus and cortex (*n* = 16 old mice; Fig. 5*A*). As expected, no plaques were detected in WT mice (*n *= 16 old mice; Fig. 5*B*). In these sections, the antibody to BDNF was used and then tissue sections were stained for thioflavin-S to identify plaques (see Materials and methods). The results are consistent with a previous study conducted in the APP23 mouse model of AD, where BDNF mRNA and Congo Red (which can be used as a marker of plaques; (Cohen and Connors, 1987) were double-labeled in cortex (Burbach et al., 2004).

These results suggest that in the MFs, BDNF protein levels are similar in WT and Tg2576 mice. However, BDNF protein is abnormal in extracellular plaques.

### MF BDNF expression in female WT and Tg2576 mice is related to estrous cycle phase

The analysis above pooled all female mice, regardless of the stage of the estrous cycle. However, previous work has shown that rat MF BDNF rises at as estrogen increases on proestrous morning, remains elevated the next morning (estrous morning) and then returns to the baseline during the subsequent days of the estrous cycle, diestrus 1 and diestrus 2 (Scharfman et al., 2003).Therefore, we reexamined MF BDNF-ir in a cohort of females where we estimated the cycle stage at the time of perfusion (young *n* = 4, old *n* = 4; WT *n* = 4, Tg2576 *n* = 4). Perfusion was done in the morning (10:00 A.M. to 12:00 P.M.). The vaginal sample was taken immediately afterwards. If a vaginal sample had primarily leukocytes, the cycle stage at the time of death was estimated to be diestrous1 or diestrous 2 morning. If epithelial or cornified epithelial cells dominated, the stage was estimated to be proestrous or estrous morning.

**Figure 6.**
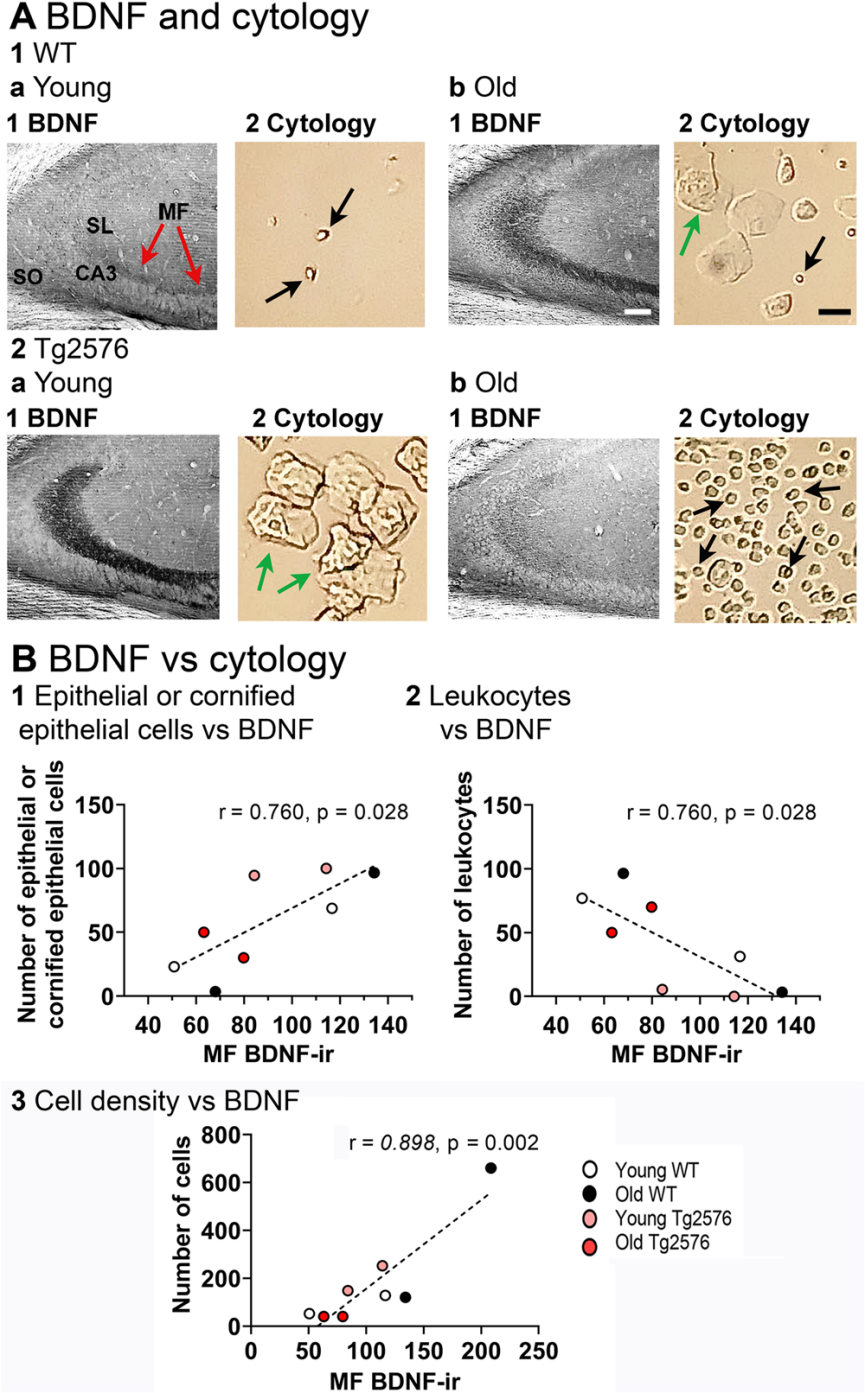
Correlation of MF BDNF protein with estrous cycle phase. ***A***, Examples of BDNF-ir and the vaginal cytology from the same mouse, sampled when the mouse was perfused. 1. WT mice. a. Young mice. 1-2. BDNF-ir was low when there were predominantly leukocytes in the vaginal sample. An example is from a young WT mouse (2 months-old), but it was found in other experimental groups also. Red arrows point to the MFs. Black arrows point to leukocytes. The calibration bar for all parts of the figure is shown in 1b. For BDNF-ir it corresponds to 100 µm; for Cytology, 25 µm. SL, stratum lucidum; SO, stratum oriens, MF, mossy fiber. b. Old mice. 1-2. An example of higher BDNF-ir in MFs in a mouse with a vaginal sample that had cornified epithelial cells (green arrow). The example is from an old WT mouse (15 months-old), but it was found in other experimental groups also. 2. Tg2576 mice. a. Young mice. 1-2. An example of BDNF-ir in a mouse with predominantly cornified epithelial cells. The example is from a young Tg2576 mouse (2 months-old), but it was found in other experimental groups also. b. Old mice. 1-2. BDNF-ir in a 14.2 months-old mouse with primarily leukocytes in the vaginal sample. This pattern was also found in other experimental groups. ***B***, BDNF-ir is correlated with cell type and cell density. 1. BDNF-ir was higher when epithelial and cornified epithelial cells were numerous (Pearson’s *r* = 0.760, *p* = 0.028 young, *n* = 4; old, *n* = 4; WT, *n* = 4; Tg2576, *n* = 4). 2. BDNF-ir was lower when leukocytes were numerous (Pearson’s *r* = 0.760, *p* = 0.028 young, *n* = 4; old, *n* = 4; WT, *n* = 4; Tg2576, *n* = 4). These data are consistent with previous studies showing that MF BDNF-ir is lower on diestrous 1 morning in the normal female rat (Scharfman et al., 2003). 3. BDNF-ir was low when the total number of cells was low (Pearson’s *r* = 0.898, *p* = 0.002). There were 8 mice for this analysis and ages were pooled (young, *n* = 4; old, *n* = 4; WT, *n* = 4; Tg2576, *n* = 4). The data are consistent with the idea that there is lower BDNF when estrous cycles are not robust.

The results showed that MF BDNF-ir varied with estimated cycle stage (Fig. 6). Quantification of cells in the vaginal sample showed a significant correlation between the type of cells and the intensity of MF BDNF-ir. When leukocytes were numerous, BDNF-ir was relatively low, and when epithelial or cornified epithelial cells were dominant, BDNF-ir was higher (Pearson’s; *r* = 0.760, *p* = 0.028; Fig. 6*B*1,2). The results are consistent with an increase in MF BDNF after the proestrous morning surge in estrogen, as shown previously for normal rodents (Scharfman et al., 2003) . Interestingly, the correlative data did not show any major differences between genotypes or age (Fig. 6). MF BDNF-ir was weak when the cell density was low (Pearson’s; *r* = 0.898, *p* = 0.002; Fig. 6*B*3). This result could be due to several reasons. First, in 2months-old females, there may not be a well-established cycle yet. Puberty typically ends at 2 months of age so estrous cycles may not be robust at this age. A low density in old females may reflect mice entering reproductive senescence. In other mice, there may be low cellularity for other reasons (Li et al., 2018).

### ΔFosB protein expression in GCs correlates with MF BDNF protein expression

ΔFosB is part of the immediate early gene protein family that is induced by neuronal activation (Minatohara et al., 2015). Its long half-life allows it to accumulate in active cells for 1–2 weeks (Nestler et al., 2001; McClung et al., 2004). In AD, ΔFosB has been shown postmortem and in murine models with mutations in APP that simulate familial AD (Chen et al., 1997, 2000; Corbett et al., 2017; You et al., 2017, 2018). These data are consistent with the evidence that there is hyperexcitability in AD and in the mouse models (Palop and Mucke, 2010; Chin and Scharfman, 2013; Vossel et al., 2017).

**Figure 7.**
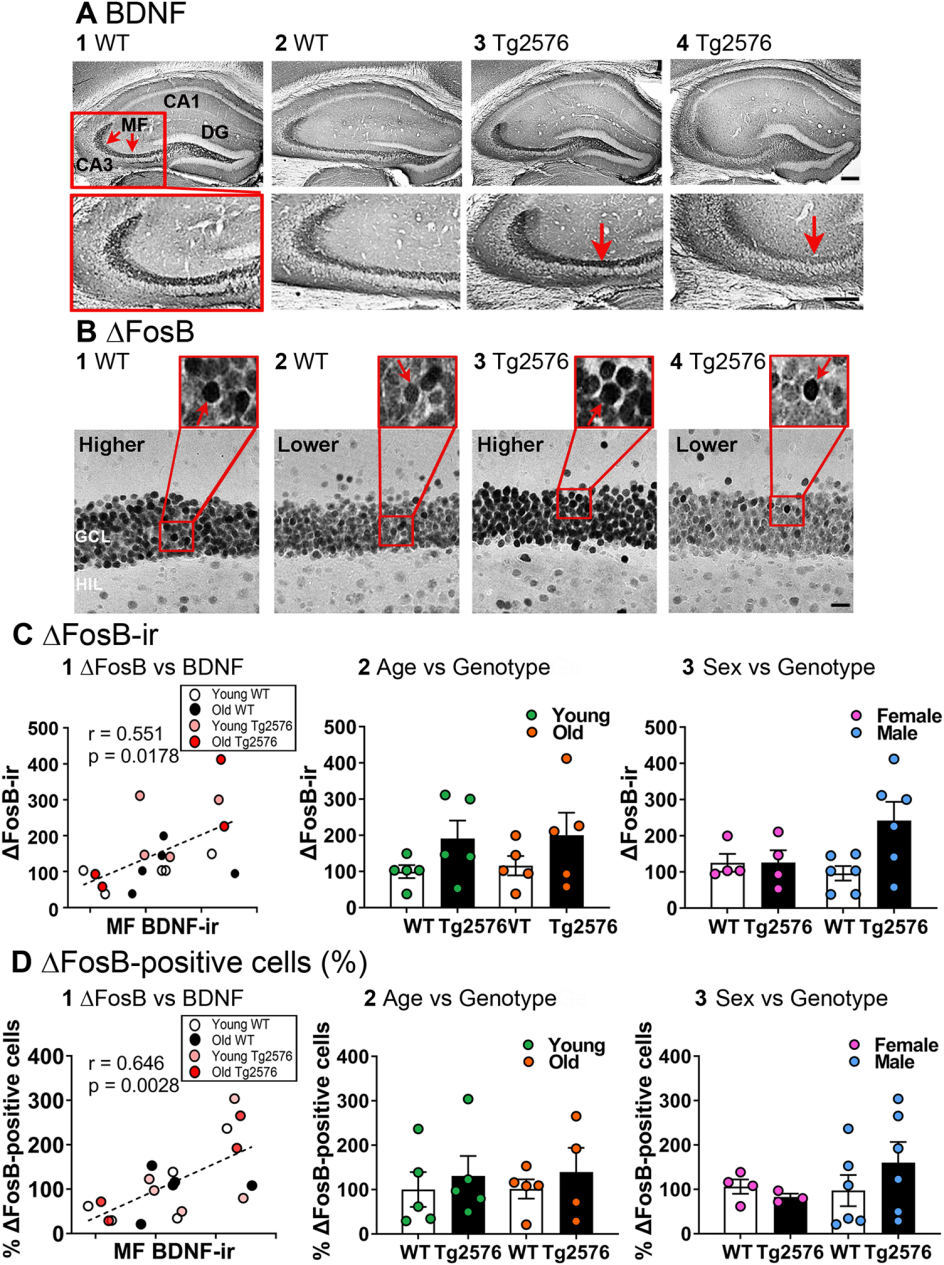
ΔFosB protein is correlated with MF BDNF protein. ***A, B***, Two examples of WT and two examples of Tg2576 mice are shown, stained to display BDNF-ir (***A***) and ΔFosB-ir (***B***) in the same mice. ***A***, Representative examples of MF BDNF-ir (red arrows) in coronal sections of dorsal hippocampus. 1-2. WT mice. 1. An example from a young (3.5 months-old) mouse. Red arrows mark BDNF-ir in MFs. The box outlined in red at the top is expanded at the bottom. 2. A section from an old (11.2 months-old) mouse. 3-4. Tg2576mice. 3. An example from a young (3.5 months-old) mouse. 4. An example from an old (11.2 months-old) mouse. Note in this example the MFs appear to exhibit less BDNF-ir than the young mouse when one examines area CA3 (red arrow). The relatively weak staining of CA3 MFs in this Tg2576 mouse was not a consistent finding. Calibration is shown in A4. Top, 200 µm; Bottom, 100 µm. ***B***, In the same mice as those presented in A, ΔFosB -ir in the GCL is shown. Calibration is in B4 and is 20 µm. Insets show ΔFosB-ir is in many GCs (red arrows). Note that lower MF BDNF correlated with lower ΔFosB-ir and higher MF BDNF with higher ΔFosB-ir. 1-2. Same WT mice as A1-2. 3-4. Same Tg2576 mice as A3-4. GCL, granule cell layer; HIL, hilus. ***C***, ΔFosB-ir in the GCL in coronal sections. 1. Correlation of MF BDNF-ir and ΔFosB -ir in the GCL. Higher levels of MF BDNF-ir were correlated with increased ΔFosB-ir in the GCL (Pearson’s *r* = 0.551, *p* = 0.018), indicating increased neuronal activity in GCs is related to BDNF protein expression in the GC axons. 2. There were no significant differences in the mean ΔFosB-ir in the GCL. A two-way ANOVA was conducted with genotype and age as factors. There was no significant effect of age (*F*_(1,16)_ = 0.091, *p* = 0.767 or genotype (*F*_(1,16)_ = 4.143, *p* = 0.059) on ΔFosB-ir. 3. To analyze sex, a Kruskal-Wallis test was conducted. There were no significant differences among the groups (*H* = 5.020, *p* = 0.170). ***D***, The number of ΔFosB-ir GCs in coronal sections. 1. Correlation of MF BDNF-ir and the number ΔFosB-ir GCs. Higher levels of MF BDNF-ir were correlated with increased numbers of GCs expressing ΔFosB protein (Pearson’s, *r*^ ^= 0.646, *p* = 0.003). 2. A two-way ANOVA was conducted with genotype and age as factors. There was no significant effect of age (*F*_(1,15)_ = 0.016, *p* = 0.902) or genotype (*F*_(1,15)_ = 0.715, *p* = 0.411) on ΔFosB-ir. 3. To analyze sex, a two-way ANOVA was conducted with sex and genotype as factors. There was no effect of sex (*F*_(1,15)_ = 0.745, *p* = 0.402) or genotype (*F*_(1,15)_ = 0.251, *p* = 0.623) on ΔFosB-ir. See Table 7-1 for more details.

Figure 7*A*,*B* shows the correlation between MF BDNF protein and ΔFosB-ir in GCs. Higher levels of BDNF protein in the MFs were significantly correlated with high GC ΔFosB-ir (Pearson’s, *r* = 0.551, *p* = 0.018; Fig. 7*C*1). The results suggest that when GC activity was high, MF BDNF protein increased independent of genotype and sex. However, when a two-way ANOVA was conducted with genotype and age as factors, there was no significant effect of genotype (*F*_(1,16)_ = 4.143, *p* = 0.059) or age (*F*_(1,16)_ = 0.091, *p* = 0.767; Fig. 7*C*2) on the mean ΔFosB-ir. Similarly, no differences in the means were found when genotype and sex were compared (Kruskal–Wallis test, *H* = 5.020, *p* = 0.170, male *n* = 6/group, female *n* = 4/ group; Fig. 7*C*3).

10.1523/ENEURO.0192-23.2023.t7-1 Extended Data Table 7-1 Normality and homogeneity of variance assessment for Figure 7, where GC ΔFosB-ir was quantified. Download Extended Data Table 7-1, DOC file.

To analyze the data with a second method, we quantified the number of GCs expressing ΔFosB-ir (Fig. 7*D*). The results were consistent with the analysis of the GCL ΔFosB-ir using thresholding. MF BDNF protein was positively correlated with the number of ΔFosB-ir GCs (Pearson’s, *r* = 0.646, *p* = 0.003; Fig. 7*D*1). However, the means were not different between groups. A two-way ANOVA conducted with genotype and age as factors confirmed no significant effect of genotype (*F*_(1,15)_ = 0.715, *p* = 0.411) or age (*F*_(1,15)_ = 0.016, *p* = 0.902; Fig. 7*D*2) on mean number of ΔFosB-ir cells. Similarly, no differences were found when genotype and sex were factors (two-way ANOVA, genotype: *F*_(1,15)_ = 0.251, *p* = 0.623; sex: *F*_(1,15)_ = 0.745, *p* = 0.402; Fig. 7*F*D2). The results for tests of normality and homogeneity of variance are shown in Extended Data Table 7-1.

### Aβ expression in different hippocampal subfields

#### Aβ is greater in area CA1 pyramidal cells of Tg2576 mice compared to WT

**Figure 8.**
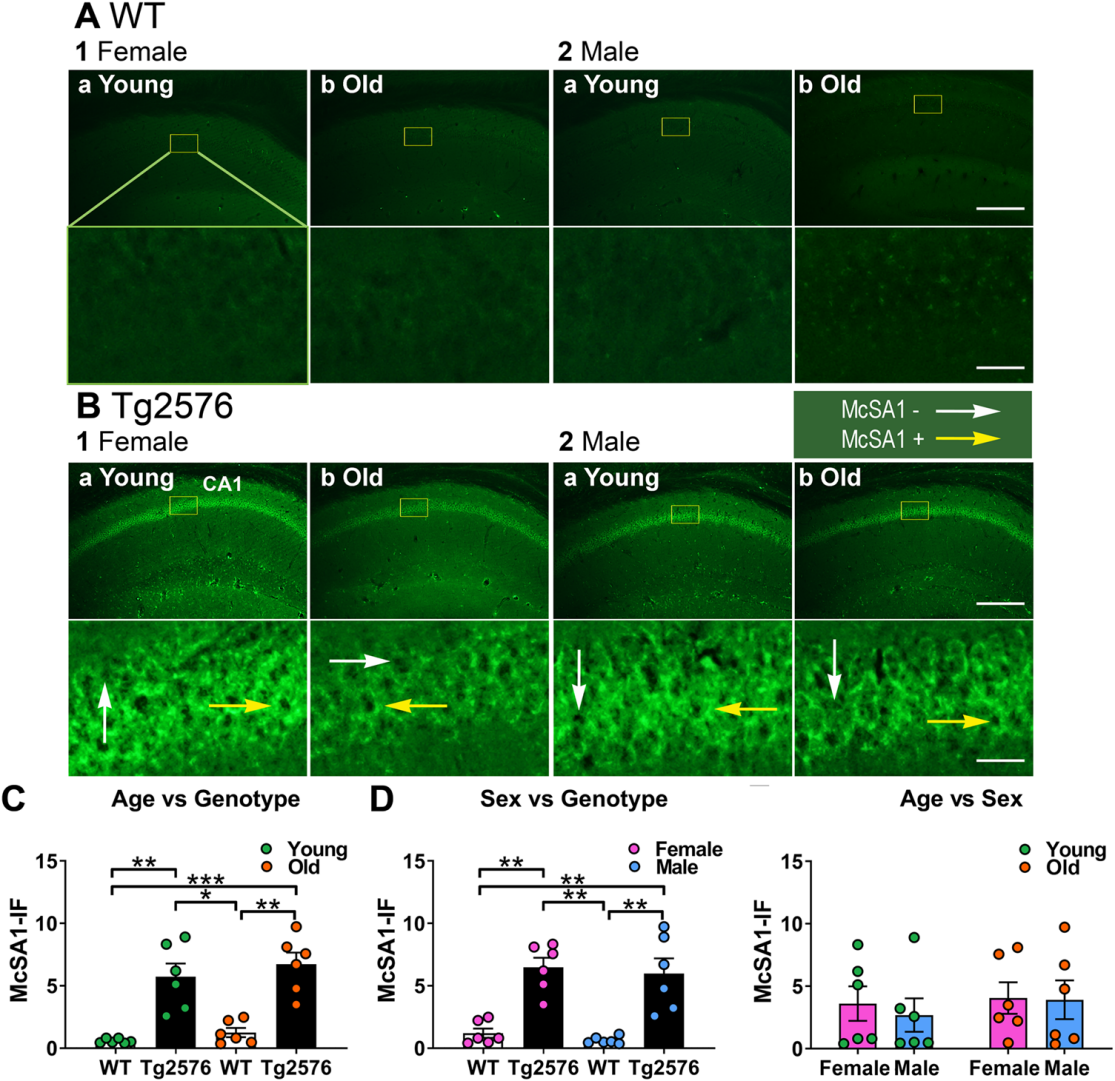
Oligomeric amyloid-β (Aβ) is greater in Tg2576 CA1 than WT in both sexes. ***A***, WT mice. Representative examples of oligomeric Aβ expression in CA1 in coronal sections of dorsal hippocampus revealed by McSA1 staining. As would be predicted in WT mice, low oligomeric Aβ expression was found in all mice. 1. Female mice. a. Young mice (2.2 months-old). Under the image is an expansion of the area surrounded by a yellow box. Calibration is shown in 2b. Top, 250 µm, Bottom, 25 µm. b. Old mice (11 months-old). 2. Male mice. a. Young mice (3.4 months-old). b. Old mice (11 months-old). ***B***, Tg2576 mice. High levels of oligomeric Aβ expression were found. 1. Female mice. a. A section from a young (3.3 months-old) mouse. Under the image is an expansion of the area surrounded by a yellow box. White arrows indicating McSA1-IF negative cells, yellow arrows indicating McSA1-IF positive cells. b. A section from an old (15.1 months-old) mouse. 2. Male mice. a. A section from a young (3.2 months-old) mouse. b. A section from an old (12 months-old) mouse. ***C***, There was significantly more oligomeric Aβ expression in CA1 of Tg2576 mice compared with WT. This difference was present at young and old ages (Kruskal-Wallis test, *H* = 17.81, *p* = 0.0005) followed by Dunn’s multiple comparisons tests (all *p* < 0.05). ***D***, In both female and male Tg2576 mice there was a significant difference in oligomeric Aβ compared to WT (Kruskal-Wallis test, *H* = 17.47, *p* = 0.0006) followed by Dunn’s multiple comparisons tests (all *p* < 0.05). ***E***, Oligomeric Aβ levels of Tg2576 mice were not dependent on age or sex (genotypes pooled, Kruskal-Wallis test, *H* = 0.673, *p* = 0.879). See Table 8-1 for more details.

One would expect greater Aβ in area CA1 pyramidal cells of Tg2576 mice relative to WT and this is what we found (Fig. 8*A*,*B*). McSA1 antibody was used to detect Aβ because it detects intracellular (oligomeric) Aβ. The difference between Tg2576 and WT mice was present at both ages 2–3 and 11–17 months (Kruskal–Wallis test, *H* = 17.81, *p* = 0.0005, *n* = 6/group; Fig. 8*C*). Both female and male Tg2576 mice showed significantly more Aβ compared to WT (Kruskal–Wallis test, *H* = 17.47, *p* = 0.0006, *n* = 6/group; Fig. 8*D*). To determine if sex differences would be significant if analyzed according to age, genotypes were pooled, and a Kruskal–Wallis test was performed with age and sex as factors. There were no significant effects (*H* = 0.673, *p* = 0.879, *n* = 6/group; Fig. 8*E*). The results for tests of normality and homogeneity of variance are shown in Extended Data Table 8-1.

10.1523/ENEURO.0192-23.2023.t8-1 Extended Data Table 8-1 Normality and homogeneity of variance assessment for Figure 8, where CA1 McSA1-IF was quantified. Download Extended Data Table 8-1, DOC file.

#### Surprisingly, GC Aβ expression is similar in Tg2576 and WT mice

**Figure 9.**
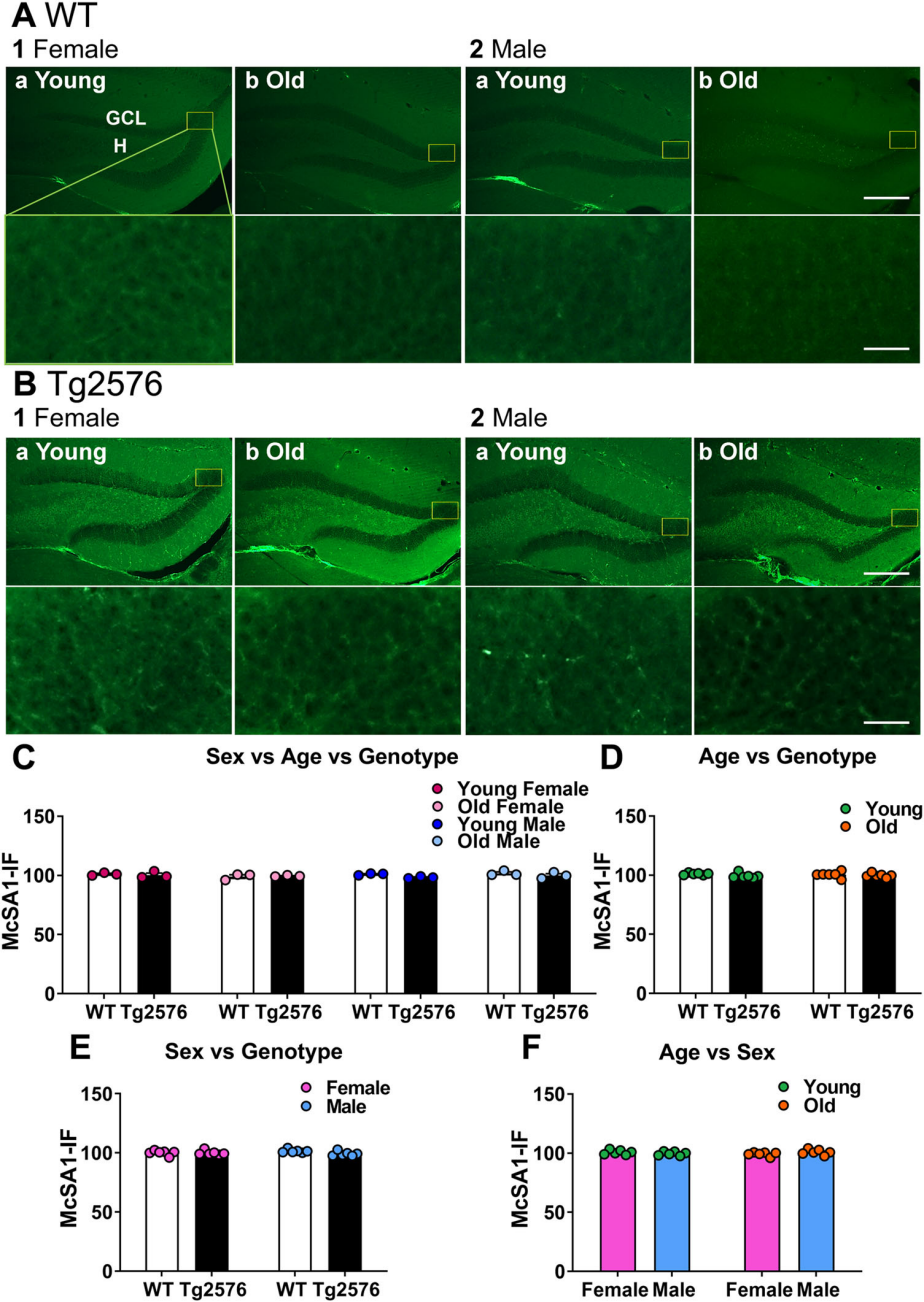
Oligomeric Aβ is low in Tg2576 GCs even at older ages. ***A***, WT mice. Representative examples of McSA1 staining in the GCL of coronal sections from dorsal hippocampus. As one would anticipate from WT mice, expression was hard to detect. 1. Female mice. a. A section from a young (2.2 months-old) mouse. Under the image is an expansion of the area surrounded by a yellow box. Calibration is shown in 2b. Top, 250 µm, Bottom, 25 µm. GCL, granule cell layer; H, hilus. b. A section from an old (16 months-old) mouse. 2. Male mice. a. A section from a young (3.4 months-old) mouse. b. A section from an old (12.3 months-old) mouse. ***B***, Tg2576 mice. McSA1 staining was hard to detect in the GCL of all mice. 1. Female mice. a. A section from a young (2.2 months-old) mouse. b. A section from an old (14.6 months-old) mouse. 2. Male mice. a. A section from a young (2.3 months-old) mouse. b. A section from an old (15.1 months-old) mouse. ***C***, There was no significant effect of genotype, age or sex on McSA1 expression (Kruskal-Wallis test, *H* = 8.600, *p* = 0.283). ***D***, There also was no significant effect of genotype or age when sexes were pooled (Kruskal-Wallis test, *H* = 5.380, *p* = 0.146). ***E***, There was no effect of genotype or sex when ages were pooled (Kruskal-Wallis test, *H* = 6.100, *p* = 0.107). ***F***, There was no effect of age or sex when genotypes were pooled (two-way ANOVA, age: *F*_(1,20)_ = 0.019, *p* = 0.892; sex: *F*_(1,20)_ = 0.104, *p* = 0.751). See Table 9-1 for more details.

We next turned to the DG. Surprisingly, McSA1-IF was not elevated in Tg2576 GCs relative to WT (Fig. 9). When a Kruskal-Wallis test was conducted with genotype and age as factors, there was no significant effect of genotype (*H* = 5.380, *p* = 0.146, *n* = 6/group; Fig. 9*D*). Similarly, there were no significant effects of genotype or sex (Kruskal-Wallis test, *H* = 6.100, *p* = 0.107, *n* = 6/group; Fig. 9*E*). To investigate age and sex differences further we pooled genotypes and found no effects (two-way ANOVA, age: *F*_(1,20)_ = 0.019, *p* = 0.892; sex: *F*_(1,20)_ = 0.104, *p* = 0.751; Fig. 9*F*). The results were the same by a Kruskal-Wallis test with genotype, age and sex as factors (*H* = 8.600, *p* = 0.283, *n* = 3/group; Fig. 9*C*). The results for tests of normality and homogeneity of variance are shown in Extended Data Table 9-1.

10.1523/ENEURO.0192-23.2023.t9-1 Extended Data Table 9-1 Normality and homogeneity of variance assessment for Figure 9, where DG McSA1-IF was quantified. Note that for a three-way ANOVA (F in the Table), normality evaluation was not possible because the n was 3/group. Therefore, a non-parametric test was used for statistical comparisons. Download Extended Data Table 9-1, DOC file.

**Figure 10.**
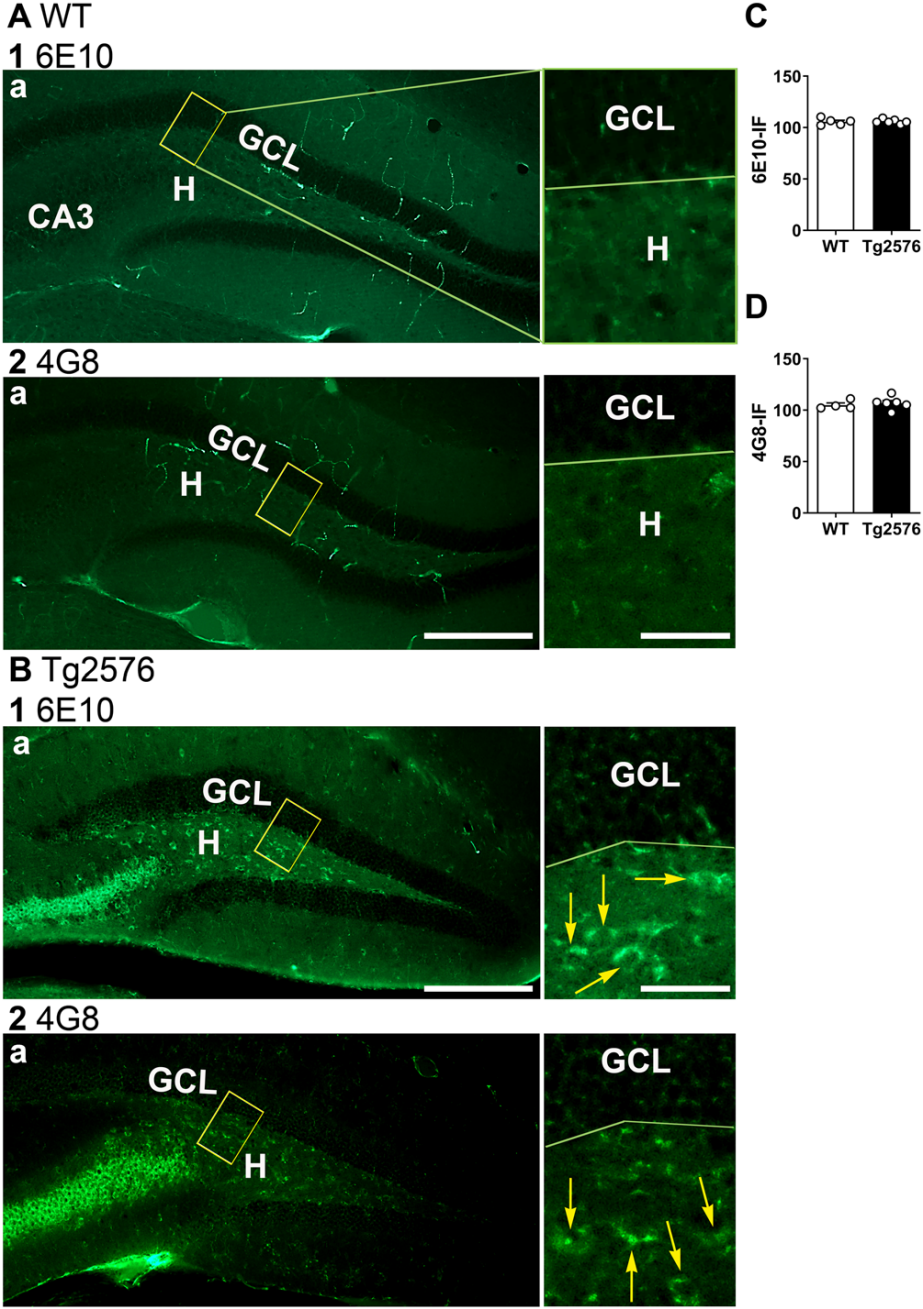
Confirmation of low GC Aβ expression using additional Aβ antibodies. ***A***, WT mice (11 months-old). Representative examples of Aβ-IF in the GCL in coronal sections of dorsal hippocampus. 1. 6E10 antibody. a-b. Weak staining is shown at low power in a. The yellow box is expanded in b. 2. 4G8 antibody. a-b. Weak staining is shown at low power in a. The yellow box is expanded in b. Calibration is shown in 2a. Top, 250 µm, Bottom, 25 µm. GCL, granule cell layer; H, hilus. ***B***, Tg2576 mice (12 months-old). Representative examples of coronal sections of dorsal hippocampus. 1. 6E10 antibody. a-b. Robust staining is shown in CA3 and the hilus but not the GCL. 2. 4G8 antibody. a-b. Strong staining is shown in CA3 and the hilus but not the GCL. Yellow arrows indicate 6E10-IF and 4G8-IF. Calibration is shown in 1a. Top, 250 µm, Bottom, 25 µm. ***C***, 6E10. There were no significant differences in Aβ-IF between WT and Tg2576 mice (sexes pooled; unpaired *t* test, *p* = 0.878, *t* = 0.158, df = 9). ***D***, 4G8. There were no significant differences in the Aβ-IF in WT and Tg2576 mice (sexes pooled, unpaired *t* test, *p* = 0.559, *t* = 0.563, df = 8). See Table 10-1 for more details.

To confirm the results with the McSA1 antibody, we used two additional antibodies to the N-terminal epitope of Aβ (6E10 and 4G8; Fig. 10*A,B*). The results confirmed that Tg2576 and WT mice were not significantly different (unpaired *t* test; 6E10, *p* = 0.878, *t* = 0.158, df = 9; 4G8, *p* = 0.589, *t* = 0.563, df = 8; Fig. 10*C,D*). The results for tests of normality and homogeneity of variance are shown in Extended Data Table 10-1.

10.1523/ENEURO.0192-23.2023.t10-1 Extended Data Table 10-1 Normality and homogeneity of variance assessment for Figure 10, where 6E10-IF and 4G8-IF were quantified. Download Extended Data Table 10-1, DOC file.

#### Tg2576 hilar cells exhibit robust Aβ although GCs do not

**Figure 11.**
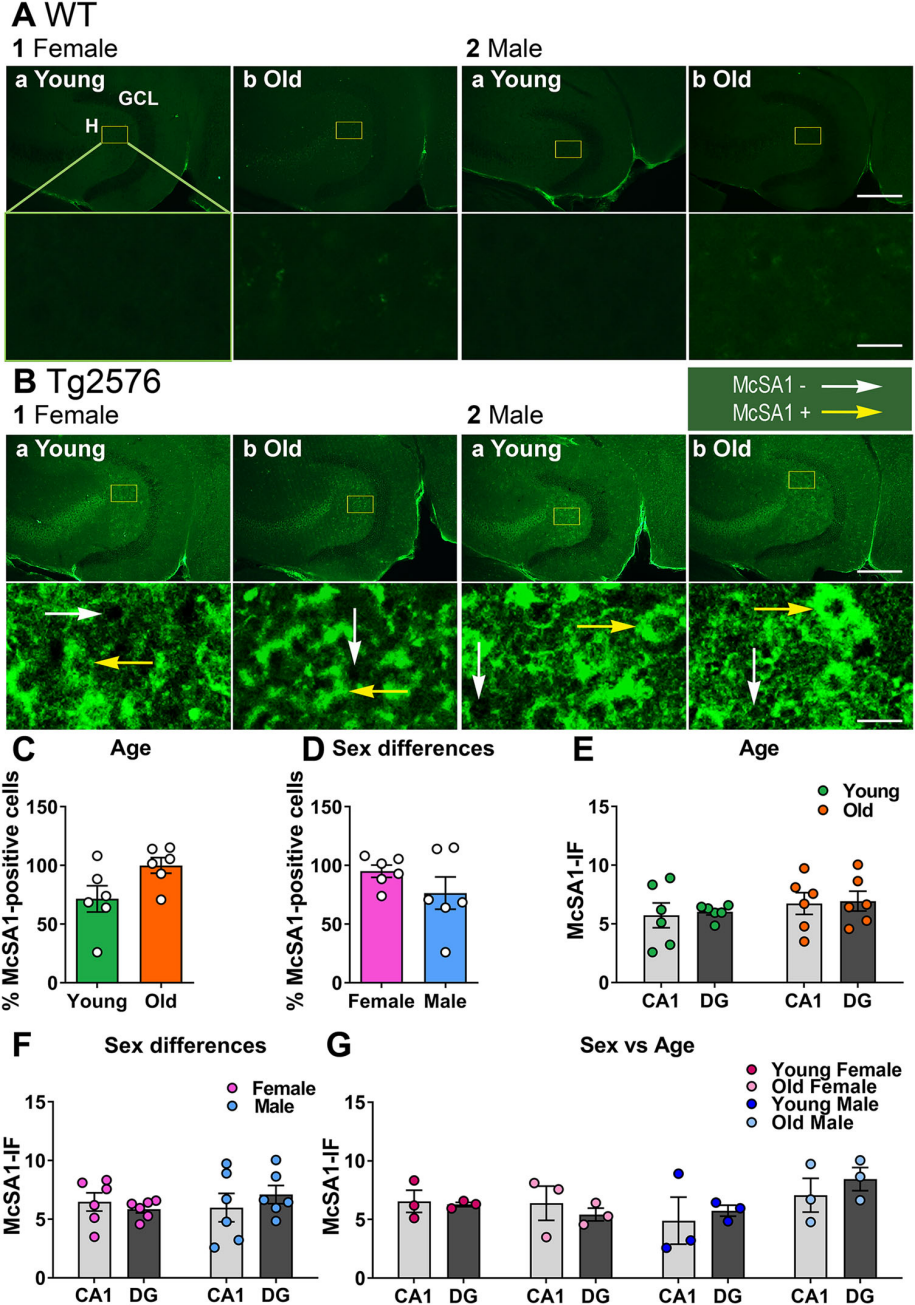
Aβ is elevated in Tg2576 hilar cells. ***A***, WT mice. Representative examples of McSA1 expression in hilar cells in horizontal sections of hippocampus. 1. Female mice. Young (a, 2.2 months-old) and old (b, 16 months-old) examples. 2. Male mice. Young (a, 3.4 months-old) and old (b, 12.3 months-old) examples. Calibration is shown in 1b. Top, 250 µm, Bottom, 25 µm. GCL, granule cell layer; H, hilus. ***B***, Tg2576 mice. Representative examples of McSA1 expression in hilar cells of horizontal sections of ventral hippocampus. Intense Aβ-IF were found in all mice. White arrows indicate McSA1-negative cells, yellow arrows indicate McSA1-positive cells. 1. Female mice. a. Young mice (2.2 months-old). The yellow box in the top image is expanded in the lower image. Calibration is shown in 1b. Top, 250 µm, Bottom, 25 µm. b. Old mice (14.6 months-old). 2. Male mice. a. Young mice (2.3 months-old). b. Old mice (15.1 months-old). ***C***, There were no differences in Aβ-IF when young and old Tg2576 mice were compared (unpaired *t* test, *p* = 0.054, *t* = 2.177, df = 10). For this comparison sexes were pooled. ***D***. To examine a sex difference, ages were pooled. There was no sex difference in Aβ-IF of Tg2576 mice (unpaired *t* test, *p* = 0.235, *t* = 1.265, df = 10). ***E***, CA1 versus hilus. A two-way ANOVA with location and age as factors showed no effect of location (*F*_(1,20)_ = 0.087, *p* = 0.771) and no effect of age (*F*_(1,20)_ = 1.344, *p* = 0.260). ***F***, To ask whether one sex might show an effect of location, a two-way ANOVA with location and sex as factors was conducted, and there was no effect of sex (*F*_(1,20)_ = 0.211, *p* = 0.651). Similar to (***E***), there was no effect of location (*F*_(1,20)_ = 0.087, *p* = 0.771). ***G***, To take all factors into account independently, a Kruskal-Wallis test was conducted. There were no effects (*H* = 6.573, *p* = 0.475). See Table 11-1 for more details.

Interestingly, hilar cells showed robust McSA1-IF in Tg2576 mice even though adjacent GCs did not. WT mice did not show any Aβ expression in the hilus (Fig. 11*A,B*). In Tg2576 mice, there was no significant effect of age on the numbers of McSA1-positive cells (unpaired *t* test, *p* = 0.054, *t* = 2.177, df = 10; Fig. 11*C*) or sex (unpaired *t* test, *p* = 0.235, *t* = 1.265, df = 10; Fig. 11*D*).

10.1523/ENEURO.0192-23.2023.t11-1 Extended Data Table 11-1 Normality and homogeneity of variance assessment for Figure 11, where horizontal DG McSA1-IF and DG/CA1 McSA1-IF were compared. Note that for a three-way ANOVA (G in the Table), normality evaluation was not possible because the n was 3/group. Therefore, a non-parametric test was used for statistical comparisons. Download Extended Data Table 11-1, DOC file.

Because hilar Aβ-IF was so strong (Fig. 11*A,B*), we compared CA1 and the hilus to determine if hilar cells had the highest Aβ-IF. To this end, the most intense IF in the CA1 cells was compared to the highest intensity for hilar cells, normalized to background in each case. A two-way ANOVA with location (CA1 vs hilus) and age as factors (sexes were pooled) showed no significant differences (location: *F*_(1,20)_ = 0.087, *p* = 0.771; age: *F*_(1,20)_ = 1.344, *p* = 0.260; Fig. 11*E*). When location and sex were main factors (ages were pooled), there were no significant effects either (location: *F*_(1,20)_ = 0.087, *p* = 0.771; sex: *F*_(1,20)_ = 0.210, *p* = 0.651; Fig. 11*F*). There also were no differences by Kruskal–Wallis test, (*H* = 6.573, *p* = 0.475, *n* = 3/group; Fig. 11*G*). Therefore, quantitative differences were not detected between CA1 and the hilus. The results for tests of normality and homogeneity of variance are shown in Extended Data Table 11-1.

## Discussion

### Summary

This study showed that MF BDNF protein does not exhibit a significant decline with age in Tg2576 mice. Even when plaques have accumulated, MF BDNF protein was not significantly different from pre-plaque ages. In addition, GCs showed elevated ΔFosB in Tg2576 mice, and it was correlated with MF BDNF protein. Potentially for these reasons, GCs showed resilience to accumulation of intracellular Aβ, despite robust Aβ in adjacent hippocampal neurons. These findings have important implications for human AD where vulnerability to amyloid plaques is considered a major contributor to pathophysiology.

### MF BDNF protein did not decline with age in WT or Tg2576 mice

Our immunohistochemical studies did not detect a significant difference in MF BDNF with aging in WT or Tg2576 mice. The lack of a decline in BDNF with age in WT mice is consistent with in situ hybridization histochemistry and ELISA in hippocampus of rats where a decline with age was not observed (Croll et al., 1998). Prior studies of hippocampal BDNF in WT animals come to a similar conclusion (Lapchak et al., 1993). However, other studies suggest that BDNF declines with age in humans. The human studies may not be suggesting there is a species difference, however, because the human studies examined circulating levels of BDNF or platelet BDNF concentration, unlike the rodent studies. In humans, serum BDNF declined with age (Lommatzsch et al., 2005; Ziegenhorn et al., 2007), and this was associated with hippocampal volume loss (Erickson et al., 2010). Therefore, the idea that BDNF declines with age and this predisposes an individual to reduced function is based primarily on serum and platelet-derived BDNF. However, other pools of BDNF, notably those within the DG, may be relatively stable with age.

If BDNF declines in area CA1 or the whole hippocampus in AD, why would that not be the case in the MFs? The resistance of GCs to insult and injury could be a reason (Scharfman, 1999). The robust expression of protective BDNF is a possible explanation. GC neuropeptide Y (NPY) is also neuroprotective and upregulated by activity, so it also could be involved in GC resilience (Vezzani et al., 1999; Marty, 2000). Indeed, NPY is upregulated in GCs in many AD murine models (Palop and Mucke, 2010).

Another contributing factor to the stability of MF BDNF could be that GCs are continually being born throughout life and the new neurons would add MF BDNF to the existing MF pathway. Increased neuronal activity increases adult neurogenesis of GCs (Bengzon et al., 1997; Parent et al., 1997, 1998), potentially elevating MF BDNF as new MFs with additional BDNF are added to SL. However, with age the rate of adult neurogenesis declines (Kempermann, 2015), and progenitors become depleted in AD mouse models (Fu et al., 2019).

### ΔFosB was elevated in Tg2576 GCs

In AD mouse models, there is increased excitability of GCs (Nenov et al., 2015; Kim et al., 2021; Smith et al., 2022) and GCs specifically (Minkeviciene et al., 2009; Alcantara-Gonzalez et al., 2021). When excitability is sufficiently elevated, seizures occur and the seizures increase GC ΔFosB (You et al., 2017; Fu et al., 2019; Stephens et al., 2020). Consistent with these studies, we found that Tg2576 GCs had elevated ΔFosB. Furthermore, the mice with elevated GC ΔFosB showed higher MF BDNF, and the correlation was statistically significant. Although the correlation does not prove that elevated GC activity and ΔFosB led to the increase in MF BDNF, it supports that hypothesis.

### The influence of sex on MF BDNF

Our findings add insight into the influence of estrous cycle on sex differences in AD. Indeed, according to the Alzheimer’s Association (2017), the incidence of AD is higher in women than in men. About two-thirds of persons diagnosed with AD are women; research show that they also appear to suffer a greater cognitive deterioration than men at the same disease stage (for a review, Li and Singh, 2014; Laws et al., 2018). In mouse models, female mice are typically affected more than males also (Callahan et al., 2001; Schmid et al., 2019; Poon et al., 2023). Therefore, there could have been a neuroendocrinological abnormality that contributed to the worse outcome of females compared to males. An abnormality that might have adverse effects is a loss of the ability of BDNF to increase in the MFs during the estrous cycle. It has been shown that as rodents pass through the stages of the estrous cycle, MF BDNF waxes and wanes (Scharfman et al., 2003). As females undergo the preovulatory estrogen surge, MF BDNF protein rises, and it remains high until the next morning, estrous morning (Scharfman et al., 2003, 2007; Harte-Hargrove et al., 2015). In the present study, we confirmed the correlation of estrous cycle phase in female mice with MF BDNF protein expression. Thus, a lack of a relationship between the estrous cycle and BDNF is unlikely to play a role in the pathophysiology of Tg2576 mice.

One of the limitations of our findings is that the stage of the estrous cycle when MF BDNF was examined was only estimated. One vaginal sample was taken at the time of death. One sample is not sufficient to allow one to determine cycle stage definitively because a cyclic pattern can only be ascertained by many consecutive days of assessment. On the other hand, our methods were sufficient to reproduce prior findings that had daily vaginal cytologic examination for several cycles. In other words, mice with higher MF BDNF showed a vaginal cytologic result consistent with proestrous or estrous morning. Therefore, the prediction of estrous cycle stage in the present study appeared to be a good prediction.

### Resistance of GCs to Aβ

A remarkable finding was a relative resistance of GCs to Aβ accumulation. Thus, when sections were processed with an antibody to Aβ that allows one to detect oligomeric (soluble, intracellular) forms of Aβ, as well as amyloid deposited extracellularly (McSA1 or thioflavin-S), staining was negligible in GCs. This result was not because the antibody or thioflavin-S was not able to detect intracellular or extracellular Aβ, because adjacent hilar neurons exhibited robust intracellular Aβ as did CA1. Furthermore, the findings were reproduced with two other antibodies to Aβ.

This result is important because, taken together with the neuroprotective effects of BDNF, GC resistance could be due, at least in part, to their ability to keep producing BDNF. However, the high activity of GCs in Tg2576 mice boosts production of other protective substances such as NPY, as mentioned above. The findings are important because if one can understand the resistance of GCs to Aβ accumulation it might be possible to use that knowledge to delay or prevent Aβ accumulation in vulnerable cells.

### Additional considerations

An important consideration is that the anti-BDNF antibodies could detect the precursor to BDNF, proBDNF, as well as mature BDNF. This is important because the stability of MF BDNF with age could be due to a rise in proBDNF. Another consideration is BDNF receptors. Thus, a rise in MF BDNF may not increase BDNF functionally if BDNF receptors, which include full-length TrkB and truncated isoforms of TrkB, decline. There is data in normal rats suggesting that TrkB mRNA declines with age (Croll et al., 1998). Other studies suggest that full-length TrkB changes somewhat (Lapchak et al., 1993), and truncated isoforms decline (Silhol et al., 2005). However, in AD the changes in TrkB may differ from normal aging (Ginsberg et al., 2019). Indeed, truncated isoforms increase in the 5xTg AD mouse model rather than declining (Nitzan et al., 2022).

## Conclusions

The results suggest that a decline in BDNF levels does not occur in all of the regions of the hippocampus in the Tg2576 AD mouse model. In particular, the impairments in learning and memory that involve GCs and the MFs are unlikely to be a result of reduced BDNF protein levels. Nevertheless, it is still possible that strategies to increase BDNF, which have been proposed (Nagahara et al., 2009; Rahman et al., 2023) may be therapeutic, and may be related to activity dependence, which is still understudied in AD and AD-relevant animal models.

## Synthesis

Reviewing Editor: Laura Volpicelli-Daley, The University of Alabama at Birmingham Decisions are customarily a result of the Reviewing Editor and the peer reviewers coming together and discussing their recommendations until a consensus is reached. When revisions are invited, a fact-based synthesis statement explaining their decision and outlining what is needed to prepare a revision will be listed below. The following reviewer(s) agreed to reveal their identity: Wei Li, Kate O'Neill. Note: If this manuscript was transferred from JNeurosci and a decision was made to accept the manuscript without peer review, a brief statement to this effect will instead be what is listed below.

Both reviewers found this to be a valuable study with the following comments.

This is an interesting study that provides some new observations and may have a therapeutic significance. The experimental approaches are appropriate and statistical analyses are good. However, as pointed below, there are some issues that authors need to address to improve this manuscript.

This manuscript advances the field by revealing how dentate gyrus (DG) granule cells (GCs) may be resistant to Alzheimer's Disease (AD) pathologies in the Tg2576 AD mouse model. The overall conclusions are that BDNF protein expression is stable in DG GC mossy fiber axons and that DG GCs remain healthy with age. This study not only provides a good discussion of the vast literature surrounding BDNF protein expression in Alzheimer's Disease models but also provides valuable insight into how AD-related changes to BDNF protein expression are not only brain region but also cell type specific.

Please address the following comments by both reviewers and resubmit:

Reviewer 1:

1. The authors use AD animals at ages of 2 and more than 11 months for their study. However, throughout the whole manuscript, there is no difference found between two groups. The authors should justify the use of these animals.

2. In Figure 1, the BDNF immunoreactivity in fluorescence and brightfield images from old WT or Tg2576 groups appears to be stronger, which is not consistent with the data.

3. In all these images from coronal or horizontal sections, BDNF levels seems to be different in different hippocampal areas (e.g., dorsal hippocampus and ventral hippocampus). The authors should examine BDNF levels in MFs from the dorsal to ventral region along hippocampal axis and determine if there is any difference.

5. In Figure 3, the data suggest that BDNF levels may be associated with AD plaques, but the rest manuscript demonstrates that higher BDNF levels is associated with lower level of AD A beta. The authors need to discuss this apparent contradiction.

6. In Figure 4, the authors show that BDNF levels are different at different estrous cycles. However, it is not clear if it has any pathophysiological relevance. Please discuss

7. In Figure 5, it looks like the authors only evaluate the intensity of FosB. The authors should also count the number of FosB-positive cells. Furthermore, the FosB intensity in Figure 5B seem to be higher in young animals of WT and Tg2576, which is inconsistent with their data.

8. In Figure 5C, the FosB intensity has a significant trend for young and old Tg2576 animals. They authors should describe this

Reviewer 2:

Major concerns:

(1) To bolster the assertion made via dFosB quantification in Figure 5 (that higher BDNF expression is related to increased neuronal activity), I suggest performing an additional experiment to quantify pERK-ir, since pERK is also known to be a marker of neuronal activity.

Minor concerns:

(1) I suggest changing the title for clarity. I had to read the title in its current version a few times to understand the main points. I suggest changing it to the following (or something similar): "Dentate gyrus granule cells show stability of BDNF protein expression in mossy fiber axons with age and resistance to Alzheimer's Disease neuropathology in a mouse model"

(2) The results discussed in lines 402-407 do not have a figure reference. If no figure exists with these data, I suggest making a supplementary figure. If the data are present in another figure, please add the figure reference.

(3) In Figure 3, the authors study how BDNF protein is present in hippocampal and cortical plaques in Tg2576 mice. I suggest they show wildtype staining in this figure or in a supplementary figure for comparison.

## Author Response

Response to Reviewers Below each comment is repeatedly exactly as stated by the Reviewer so they are in quotes. In the revised manuscript, revised text is shown in tracking and is highlighted. A clean copy, with track changes accepted, is also provided.

Editor: "Synthesis Statement for Author (Required):

Both reviewers found this to be a valuable study with the following comments.

This is an interesting study that provides some new observations and may have a therapeutic significance. The experimental approaches are appropriate and statistical analyses are good.

However, as pointed below, there are some issues that authors need to address to improve this manuscript." We are encouraged by the positive feedback and have attended carefully to each of the comments of the Reviewers below. "This manuscript advances the field by revealing how dentate gyrus (DG) granule cells (GCs) may be resistant to Alzheimer's Disease (AD) pathologies in the Tg2576 AD mouse model. The overall conclusions are that BDNF protein expression is stable in DG GC mossy fiber axons and that DG GCs remain healthy with age. This study not only provides a good discussion of the vast literature surrounding BDNF protein expression in Alzheimer's Disease models but also provides valuable insight into how AD-related changes to BDNF protein expression are not only brain region but also cell type specific." Thank you for the assessment and positive comments.

Reviewer 1: "1. The authors use AD animals at ages of 2 and more than 11 months for their study. However, throughout the whole manuscript, there is no difference found between two groups. The authors should justify the use of these animals." We regret that our rationale was unclear. Our goal was to address the hypothesis that BDNF protein in the mossy fibers changes with age. To address this goal, we chose a young age and an older age. The revised text that clarifies this point is on Pages 8-9, Lines 353-358 of the manuscript copy that has track changes highlighted:

We tested the hypothesis that BDNF protein expression changed with age. For this goal we investigated BDNF-ir in Tg2576 mice and in their WT littermates at two different ages, 2-3 months, and >11 months. The early age of 2-3 months was chosen because it is considered to be an age early in the development of AD-like features such as the appearance of plaques (as explained in the Introduction). The older age of >11 months was selected because plaques have developed by that age. "2. In Figure 1, the BDNF immunoreactivity in fluorescence and brightfield images from old WT or Tg2576 groups appears to be stronger, which is not consistent with the data." We thank the Reviewer for this comment because we did not realize that one photomicrograph does indeed seem to be unrepresentative. We replaced that micrograph with another that is more representative of the mean. The part of Fig. 1 that is revised is Fig. 1A2a. The Results are the same and the Figure Legend is the same.

The point of the Reviewer made us realize that our focus was on BDNF protein levels at the end of the MF plexus near CA2, where BDNF accumulates. However, readers may wonder whether the rest of the plexus changes with age, genotype, or sex. Therefore, we did an additional analysis of the entire plexus, and the approach is discussed in the revised Methods. The data are presented in a new Figure (Fig. 3). The results were the same as the initial analysis, i.e., BDNF protein does not change significantly with age, genotype or sex. The revised Methods section is on Page 7, Lines 262-282:

E. Quantification 1. BDNF There were two methods used for quantification. First, quantification of BDNF–immunoreactivity (BDNF-ir) was done as before (Skucas et al., 2013) by defining three regions of interest (ROIs) in stratum lucidum (SL), the location of the BDNF-rich MF projections. These three ROIs were at the end of the MF plexus where BDNF-ir is highest. The background value was measured from an ROI in SR, where BDNF-ir is relatively weak. BDNF-ir was calculated by subtracting the background value for optical density from the average optical density of the three ROIs in SL. Then MF BNDF-ir was normalized to the background of the same section. For the quantification, three sections were chosen that were approximately 300 µm apart. They were used to obtain an average for a given mouse. To quantify the BDNF-ir in dorsal hippocampus, the sections were chosen in the region ranging from the septal pole to the point in the rostral-caudal axis where the hippocampus begins to descend ventrally (1.34-2.18mm caudal to Bregma). Ventral sections were chosen where the outline of the DG assumes a C-shape (2.30-3.16 mm caudal to Bregma).

A second method of quantification examined the entire MF projection from the GCL to CA2. The background value was measured as described above. BDNF-ir was calculated by subtracting the background value from the value of the ROI of the MFs.

MF BDNF-ir quantification was then done as described above.

The revised results are on Page 9, Lines 380-396:

These data were confirmed with a second method to quantify BDNF-ir. We quantified BDNF-ir in coronal sections, measuring the entire MF projection from the GCL to CA2 (Fig. 3C). There were no significant differences in MF BDNF-ir related to genotype when we pooled sexes (Mann-Whitney U test, p = 0.712, WT n = 12, Tg2576 n = 12; Fig. 3A1). There was no significant effect of age when we pooled genotypes (Mann-Whitney U test, p = 0.887, young n = 12, old n = 12; Fig. 3A2). At young ages, WT and Tg2576 MF BDNF-ir were not significantly different (sexes pooled; Mann–Whitney U test, p = 0.240, young WT n = 6, young Tg2576 n = 6; Fig. 3A3a). MF BDNF protein in old Tg2576 mice was not significantly different from old WT mice (Mann–Whitney U test, p = 0.589, old WT n = 6, old Tg2576 n = 6; Fig. 3A3b).

When we focused on sex (Fig. 3B), the results did not show differences either. Thus, there were no significant effects of sex on MF BDNF protein (genotypes pooled, Mann–Whitney U test, p = 0.266, female n = 12, male n = 12; Fig. 3B1). WT and Tg2576 females were not significantly different (Mann-Whitney U test, p > 0.999, n = 6/group, Fig. 3B2a), and WT and Tg2576 males were not significantly different either (Mann-Whitney U test, p = 0.818, n = 6/group; Fig. 3B2b). There also were no significant differences in young and old females (Mann-Whitney U test, p = 0.937, young female n = 6, old female n = 6;

Fig. 3B3a) or young and old males (Mann-Whitney U test, p > 0.999, young male = 6, old male n = 6; Fig. 3B3b). "3. In all these images from coronal or horizontal sections, BDNF levels seem to be different in different hippocampal areas (e.g., dorsal hippocampus and ventral hippocampus). The authors should examine BDNF levels in MFs from the dorsal to ventral region along hippocampal axis and determine if there is any difference." This is a very good point and we have added to the revised manuscript an analysis of dorsal vs. ventral MF BDNF protein. The new data are in a new Figure (Fig. 4 of the revised manuscript).

The new results that discuss the analyses are in the Results section on Page 10, Lines 403- 412:

In Fig. 4D-F we focused on potential differences in MF BDNF protein expression in hippocampus across the septotemporal axis. Dorsal and ventral MF BDNF-ir were not significantly different when genotypes were pooled (unpaired t–test, p = 0.563, t = 0.583, df = 42, Fig. 4D). We also did additional statistics to further analyze dorsal and ventral regions. When a two-way ANOVA was conducted with genotype and area (dorsal vs. ventral) as factors, there was no significant effect of genotype (F(1,40) = 4.047, p = 0.051) or area (F(1,40) = 0.036, p = 0.553; Fig. 4E1). Similarly, no difference was found when age and area (dorsal vs. ventral) were factors (two-way ANOVA, age: F(1,40) = 0.128, p = 0.723; area:

F(1,40) = 0.325, p = 0.571; Fig. 4E2). A three way-ANOVA confirmed the lack of effects (genotype: F(1,36)= 0.560, p = 0.459, area (F(1,36) = 0.016, p = 0.899, age (F(1,36) = 0.560, p = 0.459, Fig. 4F). "5. In Figure 3, the data suggest that BDNF levels may be associated with AD plaques, but the rest manuscript demonstrates that higher BDNF levels is associated with lower level of AD A beta. The authors need to discuss this apparent contradiction." We thank the Reviewer for pointing out an apparent contradiction we had not noticed. In the revised manuscript we explain that there is no contradiction with this additional text (Page 10, Lines 425-426):

These results suggest that in the MFs, BDNF protein levels are similar in WT and Tg2576 mice. However, BDNF protein is abnormal in extracellular plaques.

We also revised the Results, so it is less likely to seem like there is a contradiction. The revised text is on Page 10, Lines 416-420:

Although these results suggested that there were no detectable abnormalities in the level of MF BDNF protein in Tg2576 mice compared to WT, there could have been abnormalities of another kind or in another brain area.

Indeed, we found BDNF-ir in and around extracellular plaques both in hippocampus and cortex (n = 16 old mice; Fig. 5A). "6. In Figure 4, the authors show that BDNF levels are different at different estrous cycles.

However, it is not clear if it has any pathophysiological relevance. Please discuss." We want to thank the reviewer for the comment and the opportunity to clarify the pathophysiological relevance. The revised text is in the section about sex differences in the Discussion (Page 14, Lines 582-599):

The influence of sex on MF BDNF Our findings add insight into the influence of estrous cycle on sex differences in AD. Indeed, according to the Alzheimer's Association (2017), the incidence of AD is higher in women than in men. About two thirds of persons diagnosed with AD are women; research shows that they also appear to suffer a greater cognitive deterioration than men at the same disease stage (see for a review Li and Singh, 2014; Laws et al., 2018). In mouse models, female mice are typically affected more than males also (Callahan et al., 2001; Schmid et al., 2019; Poon et al, 2023). Therefore, there could have been a neuroendocrinological abnormality that contributed to the worse outcome of females compared to males. An abnormality that might have adverse effects is a loss of the ability of BDNF to increase in the MFs during the estrous cycle. It has been shown that as rodents pass through the stages of the estrous cycle, MF BDNF waxes and wanes (Scharfman et al., 2003). As females undergo the preovulatory estrogen surge, MF BDNF protein rises, and it remains high until the next morning, estrous morning (Scharfman et al., 2003; Scharfman et al., 2007; Harte-Hargrove et al., 2015). In the present study, we confirmed the correlation of estrous cycle phase in female mice with MF BDNF protein expression. Thus, a lack of a relationship between the estrous cycle and BDNF is unlikely to play a role in the pathophysiology of Tg2576 mice. "7. In Figure 5, it looks like the authors only evaluate the intensity of FosB. The authors should also count the number of FosB-positive cells. Furthermore, the FosB intensity in Figure 5B seem to be higher in young animals of WT and Tg2576, which is inconsistent with their data." We have analyzed the data as suggested and added to the revised paper the results for the counts of ΔFosB-positive cells. The results are unchanged.

The revised Methods section is on Page 7, Lines 297-299: ΔFosB-ir in GCs was quantified by manually counting ΔFosB-positive cell bodies in the upper blade of DG GCL. The same threshold used to quantify ΔFosB-ir in the GCL was used to define a ΔFosB-ir positive GC.

The revised Results section discusses the new data and analyses on Page 11, Lines 478-484:

To analyze the data with a second method, we quantified the number of GCs expressing ΔFosB-ir (Fig. 7E-F). The results were consistent with the analysis of the GCL ΔFosB-ir using thresholding. A two-way ANOVA conducted with genotype and age as factors confirmed no significant effect of genotype (F(1,15) = 0.715, p = 0.411) or age (F(1,15) = 0.016, p = 0.902; Fig. 7E1). Similarly, no differences were found when genotype and sex were factors (two-way ANOVA, genotype: F(1,15) = 0.251, p = 0.623; sex: F(1,15) = 0.745, p = 0.402; Fig. 7E2).

MF BDNF protein was positively correlated with the number of ΔFosB-ir GCs (Pearson's, r = 0.646, p = 0.003; Fig. 7F).

Please note that the original Fig. 5 is Fig. 7 in the revised manuscript. The Legend of Fig. 7 is revised to describe the data and analyses. "8. In Figure 5C, the FosB intensity has a significant trend for young and old Tg2576 animals.

They authors should describe this." We are grateful for the opportunity to comment on this trend, because we had thought it was interesting, like the Reviewer. However, we had refrained from commenting because the trend was not significant statistically. We clarified the trend in the revised results (Page 11, Lines 461- 469):

Fig. 7A-B shows ΔFosB-ir in GCs. A two-way ANOVA was conducted with genotype and age as factors, there was no significant effect of genotype (F(1,16) = 4.143, p = 0.056) or age (F(1,16) = 0.091, p = 0.767; Fig. 7C1). Similarly, no difference was found when genotype and sex were factors (two-way ANOVA, genotype: F(1,16) = 3.572, p = 0.077; sex: F(1,16) = 1.251, p = 0.280; Fig. 7C2).

Notably, genotype showed a p value close to 0.05, so there appeared to be a trend for a genotype effect. This trend can be appreciated in Fig. 7C1-2 where the Tg2576 mice had a greater mean ΔFosB-ir than WT. This trend is consistent with the idea that Tg2576 mice are hyperexcitable compared to WT.

Reviewer 2:

Major concerns: "(1) To bolster the assertion made via dFosB quantification in Figure 5 (that higher BDNF expression is related to increased neuronal activity), I suggest performing an additional experiment to quantify pERK-ir, since pERK is also known to be a marker of neuronal activity." We appreciate the Reviewer's suggestion, but we do not think it would be helpful to investigate pERK-ir in the context of this study. One reason is that ERK activation is implicated in other mechanisms and does not reflect only neuronal activity. The following explains why we think ΔFosB is better. ΔFosB is an immediate early gene of the Fos family of transcription factors critical for an adaptive neuronal response to stimulation. In contrast to the other Fos family proteins, that are induced rapidly but transiently in response to a host of acute stimuli, ΔFosB is well known to be relatively stable over 10-14 days (Nestler, 2001, 2008, 2015; McClung et al., 2004). In contrast, ERK shows a shorter duration of activation that only lasts between minutes to hours (Wortzel and Seger, 2011; Kukushkin et al., 2022). In addition, ERK is not only phosphorylated after neuronal activity. For example, ERK is phosphorylated by mechanisms involved in other cellular biological functions, such as cell death, mitochondrial fission, autophagy, and senescence (Cagnol and Chambard, 2009; Cook et al., 2017; Guo et al., ;2020). Moreover, the Tg2576 mouse (and other mouse models of AD) have been shown to have upregulated pERK, but it may not be due to neuronal activity. Instead, it could be a direct effect of Aβ production (Peng at al., 2012; Dineley et al., 2001; Nand Rai et al., 2018). Due to the multifactorial activation of ERK and the alteration demonstrated in animal models of AD, which are not necessarily related to neuronal activity, we believe that the quantification of pERK-ir does not answer specifically to our question. While ΔFosB-ir is more suitable to specifically identify neurons that have had increased neuronal activity.

Minor concerns: "(1) I suggest changing the title for clarity. I had to read the title in its current version a few times to understand the main points. I suggest changing it to the following (or something similar):

Dentate gyrus granule cells show stability of BDNF protein expression in mossy fiber axons with age and resistance to Alzheimer's Disease neuropathology in a mouse model." We agree with the Reviewer and have changed the title to the one the Reviewer suggested. "(2) The results discussed in lines 402-407 do not have a figure reference. If no figure exists with these data, I suggest making a supplementary figure. If the data are present in another figure, please add the figure reference." We have added the data to a new Figure (Fig. 4A-B). The Results are the same. The Figure Legend is revised to describe the data and the analyses. "(3) In Figure 3, the authors study how BDNF protein is present in hippocampal and cortical plaques in Tg2576 mice. I suggest they show wildtype staining in this figure or in a supplementary figure for comparison." We have added the comparison to wildtype, which shows no staining. Note the old Figure was Fig. 3 and the Figure in the revised manuscript is Fig. 5. The Figure Legend is revised to describe the data and analyses. The results are discussed on Page 10, Lines 418-420:

Indeed, we found BDNF-ir in and around extracellular plaques both in hippocampus and cortex (n = 16 old mice; Fig. 5A). As expected, no plaques were detected in WT mice (n = 16 old mice; Fig. 5B).
