# Supplementary material for: Dentate Gyrus Granule Cells Show Stability of BDNF Protein Expression in Mossy Fiber Axons with Age, and Resistance to Alzheimer’s Disease Neuropathology in a Mouse Model
Source: eNeuro. 2024 Mar 1;11(3):ENEURO.0192-23.2023. doi: 10.1523/ENEURO.0192-23.2023 (PMC10913042; doi:10.1523/ENEURO.0192-23.2023)
Supplement: Extended Data Table 2-1 — Normality and homogeneity of variance assessment for Figure 2, where MF BDNF-ir was quantified. A non-parametric test was used for the statistical analysis when either the data did not fit a normal distribution or there was significant heteroscedasticity of variance. Download Extended Data Table 2-1, DOC file. [file eneuro-11-ENEURO.0192-23.2023-s001.doc]

| **Table 2-1: Fig. 2 Test for normal distribution and variance** | | | | |
| --- | --- | --- | --- | --- |
| **Fig. 2C1. Genotype** | | | | |
| ***Shapiro-Wilk test*** | **WT** | **Tg2576** | ***F test to compare variances*** | |
| W | 0.953 | 0.857 | F, DFn, Dfd | 1.447, 11, 11 |
| P value | 0.683 | 0.045* | P value | 0.550 |
| **Fig. 2C2. Age** | | | | |
| ***Shapiro-Wilk test*** | **Young** | **Old** | ***F test to compare variances*** | |
| W | 0.955 | 0.872 | F, DFn, Dfd | 1.386, 11, 11 |
| P value | 0.716 | 0.0704 | P value | 0.598 |
| **Fig. 2C3a. Young genotype** | | | | |
| ***Shapiro-Wilk test*** | **WT** | **Tg2576** | ***F test to compare variances*** | |
| W | 0.964 | 0.909 | F, DFn, Dfd | 1.043, 5, 5 |
| P value | 0.853 | 0.429 | P value | 0.965 |
| **Fig. 2C3b. Old genotype** | | | | |
| ***Shapiro-Wilk test*** | **WT** | **Tg2576** | ***F test to compare variances*** | |
| W | 0.883 | 0.770 | F, DFn, Dfd | 1.932, 5, 5 |
| P value | 0.285 | 0.031* | P value | 0.487 |
| **Fig. 2D1. Sex** | | | | |
| ***Shapiro-Wilk test*** | **Female** | **Male** | ***F test to compare variances*** | |
| W | 0.918 | 0.892 | F, DFn, Dfd | 1.331, 11, 11 |
| P value | 0.271 | 0.126 | P value | 0.643 |
| **Fig. 2D2a. Female genotype** | | | | |
| ***Shapiro-Wilk test*** | **WT** | **Tg2576** | ***F test to compare variances*** | |
| W | 0.940 | 0.866 | F, DFn, Dfd | 1.053, 5, 5 |
| P value | 0.660 | 0.212 | P value | 0.956 |
| **Fig. 2D2b. Male genotype** | | | | |
| ***Shapiro-Wilk test*** | **WT** | **Tg2576** | ***F test to compare variances*** | |
| W | 0.928 | 0.863 | F, DFn, Dfd | 1.930, 5, 5 |
| P value | 0.568 | 0.201 | P value | 0.488 |
| **Fig. 2D3a. Female age** | | | | |
| ***Shapiro-Wilk test*** | **Young** | **Old** | ***F test to compare variances*** | |
| W | 0.955 | 0.874 | F, DFn, Dfd | 1.544, 5, 5 |
| P value | 0.782 | 0.241 | P value | 0.645 |
| **Fig. 2Dba. Male age** | | | | |
| ***Shapiro-Wilk test*** | **Young** | **Old** | ***F test to compare variances*** | |
| W | 0.948 | 0.84 | F, DFn, Dfd | 1.389, 5, 5 |
| P value | 0.724 | 0.1304 | P value | 0.727 |
