# Supplementary material for: Dentate Gyrus Granule Cells Show Stability of BDNF Protein Expression in Mossy Fiber Axons with Age, and Resistance to Alzheimer’s Disease Neuropathology in a Mouse Model
Source: eNeuro. 2024 Mar 1;11(3):ENEURO.0192-23.2023. doi: 10.1523/ENEURO.0192-23.2023 (PMC10913042; doi:10.1523/ENEURO.0192-23.2023)
Supplement: Extended Data Table 3-1 — Normality and homogeneity of variance assessment for Figure 3. Which also shows quantified MF BDNF-ir. Download Extended Data Table 3-1, DOC file. [file eneuro-11-ENEURO.0192-23.2023-s002.doc]

| **Table 3-1: Fig. 3 Test for normal distribution and variance** | | | | |
| --- | --- | --- | --- | --- |
| **Fig. 3C1. Genotype** | | | | |
| ***Shapiro-Wilk test*** | **WT** | **Tg2576** | ***F test to compare variances*** | |
| W | 0.948 | 0.967 | F, DFn, Dfd | 1.265, 11, 11 |
| P value | 0.604 | 0.881 | P value | 0.703 |
| **Fig. 3C2. Age** | | | | |
| ***Shapiro-Wilk test*** | **Young** | **Old** | ***F test to compare variances*** | |
| W | 0.960 | 0.956 | F, DFn, Dfd | 1.747, 11, 11 |
| P value | 0.789 | 0.723 | P value | 0.369 |
| **Fig. 3C3a. Young genotype** | | | | |
| ***Shapiro-Wilk test*** | **WT** | **Tg2576** | ***F test to compare variances*** | |
| W | 0.903 | 0.955 | F, DFn, Dfd | 1.348, 5, 5 |
| P value | 0.391 | 0.782 | P value | 0.751 |
| **Fig. 3C3b. Old genotype** | | | | |
| ***Shapiro-Wilk test*** | **WT** | **Tg2576** | ***F test to compare variances*** | |
| W | 0.953 | 0.938 | F, DFn, Dfd | 1.776, 5, 5 |
| P value | 0.765 | 0.644 | P value | 0.544 |
| **Fig. 3D1. Sex** | | | | |
| ***Shapiro-Wilk test*** | **Female** | **Male** | ***F test to compare variances*** | |
| W | 0.933 | 0.9603 | F, DFn, Dfd | 1.722, 11, 11 |
| P value | 0.412 | 0.7883 | P value | 0.381 |
| **Fig. 3D2a. Female genotype** | | | | |
| ***Shapiro-Wilk test*** | **WT** | **Tg2576** | ***F test to compare variances*** | |
| W | 0.865 | 0.947 | F, DFn, Dfd | 2.188, 5, 5 |
| P value | 0.209 | 0.7159 | P value | 0.410 |
| **Fig. 3D2b. Male genotype** | | | | |
| ***Shapiro-Wilk test*** | **WT** | **Tg2576** | ***F test to compare variances*** | |
| W | 0.927 | 0.960 | F, DFn, Dfd | 1.952, 5, 5 |
| P value | 0.554 | 0.822 | P value | 0.481 |
| **Fig. 3D3a. Female age** | | | | |
| ***Shapiro-Wilk test*** | **Young** | **Old** | ***F test to compare variances*** | |
| W | 0.933 | 0.93 | F, DFn, Dfd | 2.178, 5, 5 |
| P value | 0.603 | 0.5802 | P value | 0.413 |
| **Fig. 3D3b. Male age** | | | | |
| ***Shapiro-Wilk test*** | **Young** | **Old** | ***F test to compare variances*** | |
| W | 0.995 | 0.8937 | F, DFn, Dfd | 1.266, 5, 5 |
| P value | 0.997 | 0.3382 | P value | 0.802 |
