# Supplementary material for: Dentate Gyrus Granule Cells Show Stability of BDNF Protein Expression in Mossy Fiber Axons with Age, and Resistance to Alzheimer’s Disease Neuropathology in a Mouse Model
Source: eNeuro. 2024 Mar 1;11(3):ENEURO.0192-23.2023. doi: 10.1523/ENEURO.0192-23.2023 (PMC10913042; doi:10.1523/ENEURO.0192-23.2023)
Supplement: Extended Data Table 4-1 — Normality and homogeneity of variance assessment for Figure 4, where >17 month-old mice and ventral MF BDNF-ir was quantified. Note that for a three-way ANOVA (F in the Table), normality evaluation was not possible because the n was 3/group. Therefore, a non-parametric test was used for statistical comparisons. Download Extended Data Table 4-1, DOC file. [file eneuro-11-ENEURO.0192-23.2023-s003.doc]

| **Table 4-1: Fig. 4 Test for normal distribution and variance** | | | | | | | | | |
| --- | --- | --- | --- | --- | --- | --- | --- | --- | --- |
| **Fig. 4C. Genotype** | | | | | | | | | |
| ***Shapiro-Wilk test*** | **WT** | | **Tg2576** | | ***F test to compare variances*** | | | | |
| W | 0.967 | | 0.993 | | F, DFn, Dfd | | | 4.221, 3, 3 | |
| P value | 0.823 | | 0.971 | | P value | | | 0.268 | |
| **Fig. 4D. Area** | | | | | | | | | |
| ***Shapiro-Wilk test*** | **Dorsal** | | **Ventral** | | ***F test to compare variances*** | | | | |
| W | 0.955 | | 0.894 | | F, DFn, Dfd | | | 1.249, 19, 23 | |
| P value | 0.346 | | 0.032 | | P value | | | 0.605 | |
| **Fig. 4E1. Genotype vs Area** | | | | | | | | | |
| ***Shapiro-Wilk test*** | | **Dorsal** | | | **Ventral** | | ***Brown-Forsythe ANOVA test*** | | |
|  |  | **WT** | | **T2576** | **WT** | **Tg2576** |  |  |  |
| W | | 0.938 | | 0.941 | 0.864 | 0.931 | F, DFn, Dfd | | 1.527, 3.000, 36.09 |
| P value | | 0.478 | | 0.508 | 0.084 | 0.460 | P value | | 0.224 |
| **Fig. 4E2. Age vs Area** | | | | | | | | | |
| ***Shapiro-Wilk test*** | | **Dorsal** | | | **Ventral** | | ***Brown-Forsythe ANOVA test*** | | |
|  |  | **Young** | | **Old** | **WT** | **Young** |  |  |  |
| W | | 0.961 | | 0.914 | 0.937 | 0.829 | F, DFn, Dfd | | 0.166, 3.000, 37.71 |
| P value | | 0.799 | | 0.242 | 0.517 | 0.032 | P value | | 0.918 |
| **Fig. 4F. Genotype vs Area vs Age** | | | | | | | | | |
| ***Brown-Forsythe ANOVA test*** | | | | | | | | | |
| F, DFn, Dfd | | | | | 0.440, 3.000, 2.323 | | | | |
| P value | | | | | 0.746 | | | | |
