# Supplementary material for: Dentate Gyrus Granule Cells Show Stability of BDNF Protein Expression in Mossy Fiber Axons with Age, and Resistance to Alzheimer’s Disease Neuropathology in a Mouse Model
Source: eNeuro. 2024 Mar 1;11(3):ENEURO.0192-23.2023. doi: 10.1523/ENEURO.0192-23.2023 (PMC10913042; doi:10.1523/ENEURO.0192-23.2023)
Supplement: Extended Data Table 7-1 — Normality and homogeneity of variance assessment for Figure 7, where GC ΔFosB-ir was quantified. Download Extended Data Table 7-1, DOC file. [file eneuro-11-ENEURO.0192-23.2023-s004.doc]

| **Table 7-1: Fig. 7 Test for normal distribution and variance** | | | | | | |
| --- | --- | --- | --- | --- | --- | --- |
| **Fig. 7C1. Genotype vs Age** | | | | | | |
| ***Shapiro-Wilk test*** | **Young** | | **Old** | | ***Brown-Forsythe ANOVA test*** | |
|  | **WT** | **T2576** | **WT** | **Tg2576** |  |  |
| W | 0.867 | 0.884 | 0.982 | 0.959 | F, DFn, Dfd | 1.686, 3.000, 7.617 |
| P value | 0.255 | 0.328 | 0.945 | 0.772 | P value | 0.250 |
| **Fig. 7C2. Genotype vs Sex** | | | | | | |
| ***Shapiro-Wilk test*** | **Female** | | **Male** | | ***Brown-Forsythe ANOVA test*** | |
|  | **WT** | **T2576** | **WT** | **Tg2576** |  |  |
| W | 0.702 | 0.981 | 0.859 | 0.977 | F, DFn, Dfd | 3.860, 3.000, 10.86 |
| P value | 0.012* | 0.909 | 0.186 | 0.934 | P value | 0.042* |
| **Fig. 7E1. Genotype vs Age** | | | | | | |
| ***Shapiro-Wilk test*** | **Young** | | **Old** | | ***Brown-Forsythe ANOVA test*** | |
|  | **WT** | **T2576** | **WT** | **Tg2576** |  |  |
| W | 0.858 | 0.797 | 0.852 | 0.940 | F, DFn, DFd | 0.231, 3.000, 11.32 |
| P value | 0.229 | 0.077 | 0.201 | 0.656 | P value | 0.873 |
| **Fig. 7E2. Genotype vs Sex** | | | | | | |
| ***Shapiro-Wilk test*** | **Female** | | **Male** | | ***Brown-Forsythe ANOVA test*** | |
|  | **WT** | **T2576** | **WT** | **Tg2576** |  |  |
| W | 0.939 | 0.958 | 0.874 | 0.932 | F, DFn, Dfd | 1.197, 3.000, 10.57 |
| P value | 0.646 | 0.608 | 0.243 | 0.598 | P value | 0.357 |
