# Supplementary material for: Dentate Gyrus Granule Cells Show Stability of BDNF Protein Expression in Mossy Fiber Axons with Age, and Resistance to Alzheimer’s Disease Neuropathology in a Mouse Model
Source: eNeuro. 2024 Mar 1;11(3):ENEURO.0192-23.2023. doi: 10.1523/ENEURO.0192-23.2023 (PMC10913042; doi:10.1523/ENEURO.0192-23.2023)
Supplement: Extended Data Table 8-1 — Normality and homogeneity of variance assessment for Figure 8, where CA1 McSA1-IF was quantified. Download Extended Data Table 8-1, DOC file. [file eneuro-11-ENEURO.0192-23.2023-s005.doc]

| **Table 8-1: Fig. 8 Test for normal distribution and variance** | | | | | | |
| --- | --- | --- | --- | --- | --- | --- |
| **Fig. 8C. Genotype vs Age** | | | | | | |
| ***Shapiro-Wilk test*** | **Young** | | **Old** | | ***Brown-Forsythe ANOVA test*** | |
|  | **WT** | **T2576** | **WT** | **Tg2576** |  |  |
| W | 0.798 | 0.930 | 0.872 | 0.974 | F, DFn, DFd | 18.18,3.000, 11.18 |
| P value | 0.057 | 0.579 | 0.234 | 0.918 | P value | 0.0001* |
| **Fig. 8D. Genotype vs Sex** | | | | | | |
| ***Shapiro-Wilk test*** | **Female** | | **Male** | | ***Brown-Forsythe ANOVA test*** | |
|  | **WT** | **T2576** | **WT** | **Tg2576** |  |  |
| W | 0.800 | 0.915 | 0.860 | 0.925 | F, DFn, DFd | 17.18, 3.000, 9.770 |
| P value | 0.059 | 0.471 | 0.190 | 0.543 | P value | 0.0003* |
| **Fig. 8E. Age vs Sex** | | | | | | |
| ***Shapiro-Wilk test*** | **Young** | | **Old** | | ***Brown-Forsythe ANOVA test*** | |
|  | **WT** | **T2576** | **WT** | **Tg2576** |  |  |
| W | 0.853 | 0.762 | 0.885 | 0.884 | F, DFn, DFd | 0.196, 3.000, 19.51 |
| P value | 0.167 | 0.026* | 0.293 | 0.289 | P value | 0.898 |
