# Supplementary material for: Dentate Gyrus Granule Cells Show Stability of BDNF Protein Expression in Mossy Fiber Axons with Age, and Resistance to Alzheimer’s Disease Neuropathology in a Mouse Model
Source: eNeuro. 2024 Mar 1;11(3):ENEURO.0192-23.2023. doi: 10.1523/ENEURO.0192-23.2023 (PMC10913042; doi:10.1523/ENEURO.0192-23.2023)
Supplement: Extended Data Table 9-1 — Normality and homogeneity of variance assessment for Figure 9, where DG McSA1-IF was quantified. Note that for a three-way ANOVA (F in the Table), normality evaluation was not possible because the n was 3/group. Therefore, a non-parametric test was used for statistical comparisons. Download Extended Data Table 9-1, DOC file. [file eneuro-11-ENEURO.0192-23.2023-s006.doc]

| **Table 9-1: Fig. 9 Test for normal distribution and variance** | | | | | | |
| --- | --- | --- | --- | --- | --- | --- |
| **Fig. 9F. Sex vs Age vs Genotype** | | | | | | |
| ***Brown-Forsythe ANOVA test*** | | | | | | |
| F, DFn, Dfd | | | 0.874, 3.000, 2.040 | | | |
| P value | | | 0.571 | | | |
| **Fig. 9D. Age vs Genotype** | | | | | | |
| ***Shapiro-Wilk test*** | **Young** | | **Old** | | ***Brown-Forsythe ANOVA test*** | |
|  | **WT** | **T2576** | **WT** | **Tg2576** |  |  |
| W | 0.945 | 0.758 | 0.846 | 0.931 | F, DFn, DFd | 0.912, 3.000, 15.28 |
| P value | 0.6971 | 0.024* | 0.146 | 0.586 | P value | 0.458 |
| **Fig. 9E. Sex vs Genotype** | | | | | | |
| ***Shapiro-Wilk test*** | **Female** | | **Male** | | ***Brown-Forsythe ANOVA test*** | |
|  | **WT** | **T2576** | **WT** | **Tg2576** |  |  |
| W | 0.839 | 0.757 | 0.839 | 0.853 | F, DFn, DFd | 1.530, 3.000, 18.74 |
| P value | 0.127 | 0.023* | 0.127 | 0.167 | P value | 0.240 |
| **Fig. 9F. Age vs Sex** | | | | | | |
| ***Shapiro-Wilk test*** | **Young** | | **Old** | | ***Brown-Forsythe ANOVA test*** | |
|  | **Female** | **Male** | **Female** | **Male** |  |  |
| W | 0.957 | 0.9397 | 0.825 | 0.977 | F, DFn, DFd | 1.000, 3.000, 18.45 |
| P value | 0.798 | 0.657 | 0.098 | 0.935 | P value | 0.4149 |
