# Supplementary material for: Dentate Gyrus Granule Cells Show Stability of BDNF Protein Expression in Mossy Fiber Axons with Age, and Resistance to Alzheimer’s Disease Neuropathology in a Mouse Model
Source: eNeuro. 2024 Mar 1;11(3):ENEURO.0192-23.2023. doi: 10.1523/ENEURO.0192-23.2023 (PMC10913042; doi:10.1523/ENEURO.0192-23.2023)
Supplement: Extended Data Table 10-1 — Normality and homogeneity of variance assessment for Figure 10, where 6E10-IF and 4G8-IF were quantified. Download Extended Data Table 10-1, DOC file. [file eneuro-11-ENEURO.0192-23.2023-s007.doc]

| **Table 10-1: Fig. 10 Test for normal distribution and variance** | | | | |
| --- | --- | --- | --- | --- |
| **Fig. 10C. 6E10 Genotype** | | | | |
| ***Shapiro-Wilk test*** | **WT** | **Tg2576** | ***F test to compare variances*** | |
| W | 0.941 | 0.934 | F, DFn, Dfd | 2.413, 4, 5 |
| P value | 0.672 | 0.610 | P value | 0.360 |
| **Fig. 10D. 4G8 Genotype** | | | | |
| ***Shapiro-Wilk test*** | **WT** | **Tg2576** | ***F test to compare variances*** | |
| W | 0.784 | 0.917 | F, DFn, Dfd | 2.143, 5, 3 |
| P value | 0.077 | 0.484 | P value | 0.563 |
