# Supplementary material for: Dentate Gyrus Granule Cells Show Stability of BDNF Protein Expression in Mossy Fiber Axons with Age, and Resistance to Alzheimer’s Disease Neuropathology in a Mouse Model
Source: eNeuro. 2024 Mar 1;11(3):ENEURO.0192-23.2023. doi: 10.1523/ENEURO.0192-23.2023 (PMC10913042; doi:10.1523/ENEURO.0192-23.2023)
Supplement: Extended Data Table 11-1 — Normality and homogeneity of variance assessment for Figure 11, where horizontal DG McSA1-IF and DG/CA1 McSA1-IF were compared. Note that for a three-way ANOVA (G in the Table), normality evaluation was not possible because the n was 3/group. Therefore, a non-parametric test was used for statistical comparisons. Download Extended Data Table 11-1, DOC file. [file eneuro-11-ENEURO.0192-23.2023-s008.doc]

| **Table 11-1: Fig. 11 Test for normal distribution and variance** | | | | | | | | | |
| --- | --- | --- | --- | --- | --- | --- | --- | --- | --- |
| **Fig. 11C. Age** | | | | | | | | | |
| ***Shapiro-Wilk test*** | **Young** | | **Old** | | ***F test to compare variances*** | | | | |
| W | 0.960 | | 0.889 | | F, DFn, Dfd | | | 2.758, 5, 5 | |
| P value | 0.818 | | 0.311 | | P value | | | 0.290 | |
| **Fig. 11D. Sex differences** | | | | | | | | | |
| ***Shapiro-Wilk test*** | **Female** | | **Male** | | ***F test to compare variances*** | | | | |
| W | 0.930 | | 0.897 | | F, DFn, Dfd | | | 6.942, 5, 5 | |
| P value | 0.577 | | 0.360 | | P value | | | 0.053 | |
| **Fig. 11E. Age** | | | | | | | | | |
| ***Shapiro-Wilk test*** | | **Young** | | | **Old** | | ***Brown-Forsythe ANOVA test*** | | |
|  |  | **CA1** | | **DG** | **CA1** | **DG** |  |  |  |
| W | | 0.930 | | 0.841 | 0.974 | 0.946 | F, DFn, DFd | | 0.478, 3.000, 15.16 |
| P value | | 0.579 | | 0.132 | 0.918 | 0.707 | P value | | 0.702 |
| **Fig. 11F. Sex differences** | | | | | | | | | |
| ***Shapiro-Wilk test*** | | **Female** | | | **Male** | | ***Brown-Forsythe ANOVA test*** | | |
|  |  | **CA1** | | **DG** | **CA1** | **DG** |  |  |  |
| W | | 0.915 | | 0.889 | 0.925 | 0.937 | F, DFn, DFd | | 0.461, 3.000, 13.36 |
| P value | | 0.471 | | 0.311 | 0.543 | 0.633 | P value | | 0.714 |
| **Fig. 11G. Sex vs Age vs Area** | | | | | | | | | |
| ***Brown-Forsythe ANOVA test*** | | | | | | | | | |
| F, DFn, Dfd | | | | | 4.760 (3.000, 2.644) | | | | |
| P value | | | | | 0.134 | | | | |
